# Supply–demand strategies for near-term climate benefits from hydrogen in the United States

**DOI:** 10.1073/pnas.2519606122

**Published:** 2025-10-06

**Authors:** Youyi Xu, Wei Peng, Yuan Yao

**Affiliations:** ^a^Center for Industrial Ecology, Yale School of the Environment, Yale University, New Haven, CT 06511; ^b^Andlinger Center for Energy and the Environment, Princeton University, Princeton, NJ 08544; ^c^School of Public and International Affairs, Princeton University, Princeton, NJ 08544; ^d^Chemical and Environmental Engineering, Yale School of Engineering and Applied Science, Yale University, New Haven, CT 06511

**Keywords:** hydrogen, life cycle assessment, integrated assessment model, bioenergy, energy policy

## Abstract

Hydrogen (H_2_) is a promising energy carrier for decarbonization. However, the greenhouse gas (GHG) mitigation potential of the H_2_ supply and its adoption across various demand sectors in the US remains unclear. Here, we couple prospective life cycle assessment with a process-based integrated assessment model to evaluate the GHG mitigation of H_2_ from supply and demand perspectives. Our results show the critical role of H_2_ production mix in determining supply-side GHG mitigation potential. Without national carbon pricing or large-scale electrolysis in the near future, biomass-based H_2_ (Bio-H_2_) emerges as a critical transitional clean H_2_ production technology. Bio-H_2_ reduces the life cycle GHG emission intensity of the H_2_ supply mix by 1.8 to 5.5 kg CO_2_ kg^−1^ H_2_, resulting in a national reduction of 606 to 1,706 Mt CO_2_e from 2025 to 2050. This represents 1.6 to 2 times greater mitigation potential compared to scenarios without Bio-H_2_. On the demand side, we identify a misalignment: industrial sectors with high mitigation intensity (e.g., iron and steel, 147 Mt CO_2_e EJ^−1^) receive limited H_2_ deployment (0.8 EJ), while the transportation sector accounts for 75% of H_2_ use (e.g., passenger and light-duty vehicles, 10 EJ) despite their lower mitigation intensities (54 Mt CO_2_e EJ^−1^). While economy-wide climate policies (e.g., carbon prices) could direct more industrial H_2_ usages, implementing these policies is challenging; sector-specific strategies can be more practical. Our results highlight the importance of near-term support for Bio-H_2_ and sector-specific demand strategies to enhance the climate effectiveness of US H_2_ deployment.

## Significance

Hydrogen (H_2_) is critical for energy decarbonization. Previous H_2_ research has overlooked key dynamics in H_2_ supply and demand. Our study reveals that the H_2_ supply mix is crucial for greenhouse gas (GHG) mitigation, with biomass-based H_2_ providing substantial reductions in the near term when carbon pricing or large-scale advanced water electrolysis is unavailable. On the demand side, our results identify a misalignment between H_2_ deployment and GHG mitigation intensity: industrial sectors show higher GHG mitigation intensity but receive limited H_2_, while transportation sectors consume most H_2_ despite lower mitigation intensity. This highlights missed opportunities for maximizing emissions reductions. Our findings underscore the need to integrate clean H_2_ production incentives with sector-specific demand-side strategies to support national decarbonization goals.

Hydrogen (H_2_) is a zero-carbon energy carrier with no carbon dioxide (CO_2_) emissions during energy conversion, making it a promising option to displace carbon-intensive fossil fuel uses. It has been proposed as a plausible complement to electrification for achieving ambitious decarbonization targets, particularly in hard-to-abate sectors without cost-effective electrification options (1, 2). The US remains a major global H_2_ producer, contributing around 10% of the global annual production (3). The US Department of Energy has set a production target to scale up clean H_2_ production to 50 million metric tons by 2050 (4). These targets align with the broader national goal of reducing greenhouse gas (GHG) emissions and achieving net-zero emissions by 2050 (5). To support this transition, the Inflation Reduction Act (IRA) has introduced the 45V clean H_2_ production tax credit, the nation’s first dedicated incentive for clean H_2_ production in 2022 (6). It provides a per-kilogram subsidy for H_2_ production based on its life cycle GHG intensity. However, the recently approved One Big Beautiful Bill Act eliminates 45V clean H_2_ credits for projects after 2027 (7), undermining the establishment of clean H_2_ infrastructure in the future. This changing policy landscape adds a layer of unpredictability to the pace and scale of H_2_ deployment. Therefore, a clear understanding of the mitigation potential and deployment challenges of the H_2_ sector in the United States remains crucial for informing decision-making and guiding future investments in H_2_.

H_2_ supply and demand collectively determine its GHG mitigation potential (8). On the supply side, current H_2_ production is predominantly carbon-intensive, relying on natural gas steam methane reforming (NG SMR) (9). Many life cycle assessment (LCA) studies have recognized electrolysis powered by renewable electricity as the lowest-emission H_2_ production option (10–12). However, scaling up electrolytic H_2_ faces barriers, including high capital costs (13), short-term constraints in electrolysis capacity expansion to meet long-term H_2_ needs (14), and limited land availability and water resources (15). The elimination of the 45V tax credits negatively impacts renewable water electrolytic H_2_, which is currently qualified for the highest tier of tax credits. Without this incentive, the development of water electrolysis may fall further behind. In contrast, biomass-based H_2_ (Bio-H_2_) is less dependent on 45V credits and has already attracted growing attention due to its technological maturity, lower production costs, potential to leverage existing bioenergy infrastructure (16, 17) and mitigate forest fire risks (18). Studies have shown that although Bio-H_2_ generally results in higher emissions than electrolytic H_2_, it still offers substantial reductions compared to fossil-based H_2_ (16, 19). Given the abundance of biomass resources (20, 21) and mature bioenergy systems (22) in the United States, Bio-H_2_ presents a viable complementary pathway to help meet domestic clean H_2_ production goals, particularly in the near term under a shifting policy environment.

On the demand side, H_2_ can be utilized across various sectors, such as steelmaking (23), transportation (24), and heating (25), each with different GHG mitigation implications. The GHG mitigation efficacy of H_2_ depends on its adoption rates in these sectors and the GHG emissions of conventional technologies it replaces. Therefore, a comprehensive understanding of both the supply and demand dynamics of H_2_ is crucial for assessing its climate benefits. Recent studies (4, 9) provide qualitative assessments of H_2_ end-use deployment. These studies prioritize H_2_ end-use sectors based on feasibility and relative importance, but they do not conduct quantitative assessments of GHG mitigation across end-use applications. Several LCA studies have quantified the mitigation potential of H_2_ technology by replacing traditional fossil-based technology in the energy (26, 27), transportation (28, 29), refining (30), and manufacturing (31–33) industries. However, they are typically limited to single-sector analyses and do not provide a system-level comparison across H_2_ end-use sectors. In addition, these studies generally assume a single source of H_2_ supply, overlooking the current and evolving mix of production technologies. Moreover, the avoided emissions are typically calculated by assuming a 1:1 energy displacement (20) based on the energy intensities of H_2_ and a representative fossil fuel. This attributional approach overlooks the market competition among all available technologies in various end-use sectors where the introduction of H_2_ displaces traditional technologies or fuels with different replacement ratios.

Notably, comprehensive GHG assessments that quantitatively consider the interactions between H_2_ supply and demand options are lacking. A few studies have integrated LCA with market-wide analysis to quantify the environmental impact of producing a given amount of H_2_ from supply side (1, 8, 19, 34, 35); however, the GHG mitigation potential across different H_2_ end-use sectors remains unclear. These studies have leveraged process-based Integrated Assessment Models (IAMs) to simulate future economy-wide changes in background processes (e.g., electricity generation and fuel production). Studies projecting future H_2_ market often focus on either costs (36) or its role in achieving net zero (35, 37), with few examining demand-side GHG mitigation in response to future changes in policy, market, and H_2_ production technologies.

We address these gaps by answering three underexplored research questions: 1) What are the effects of different policy options and technological advancements on H_2_ production and consumption? 2) How do these supply–demand responses affect life cycle GHG emissions and, more broadly, the nationwide GHG mitigation potential of H_2_? 3) What are the opportunities to further increase H_2_’s GHG mitigation potential from both supply and demand perspectives?

**Fig. 1.**
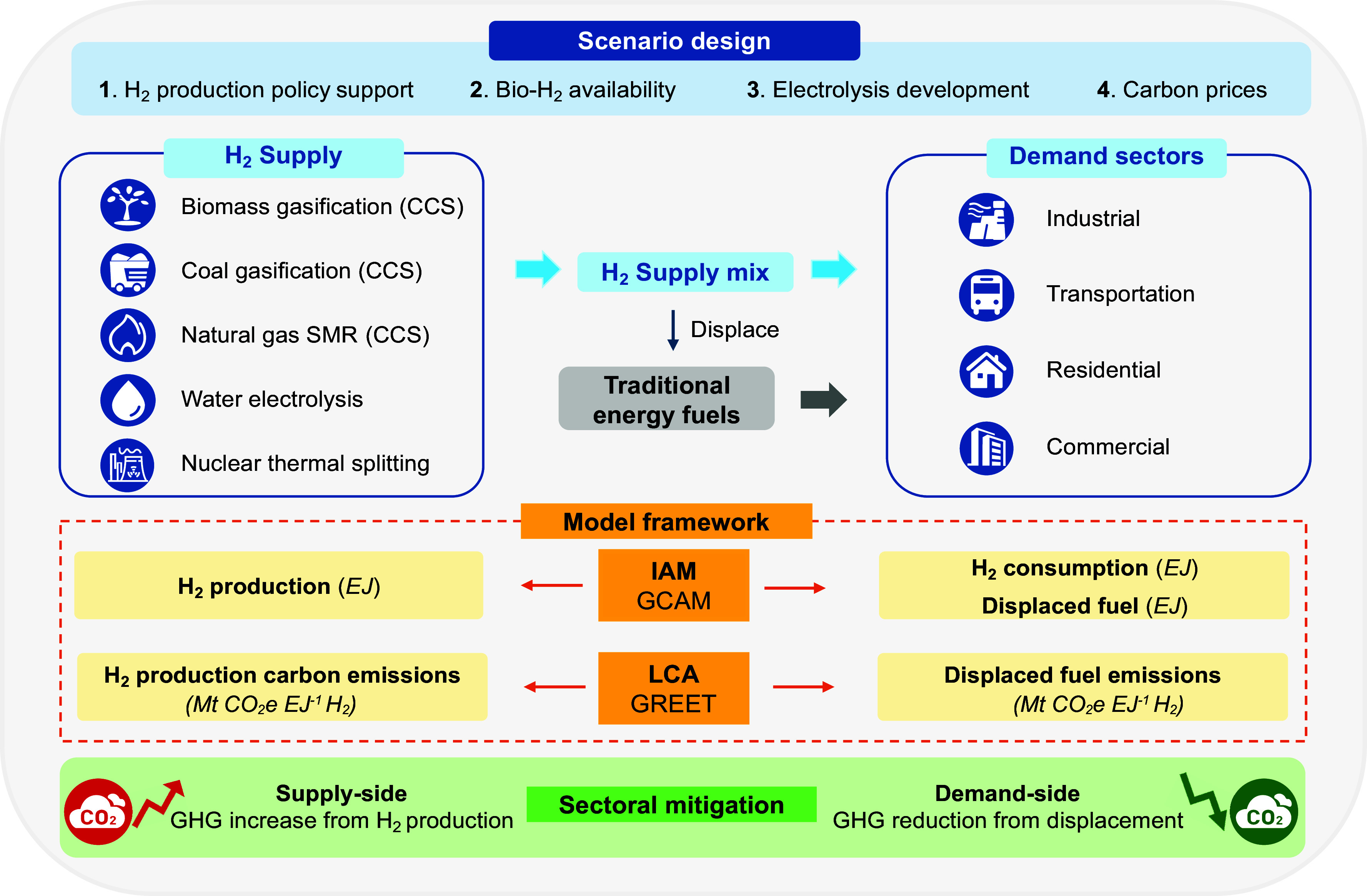
A schematic diagram of the LCA-IAM modeling framework in this study.

Addressing these questions requires new methods that can integrate energy system modeling insights into the life cycle GHG modeling. Traditional attributional LCA methods are limited in their ability to capture technology competitions and supply–demand dynamics as they are static, retrospective, and unable to capture market dynamics or system-level substitution effects. While IAMs are well suited for simulating future market responses and policy impacts, they often lack detailed representation of novel technologies and do not provide comprehensive life cycle GHG emission accounting. Here, we developed a framework that links prospective, consequential LCA with the Global Change Analysis Model (GCAM), a partial equilibrium IAM (Fig. 1). This approach allows us to combine microlevel technology details with macroscale market dynamics (38) to capture fuel substitution and technology competition. Our study presents a holistic framework for quantifying H_2_’s system-wide mitigation potential, supporting effective and targeted decarbonization strategies in the United States.

## Results

### Decarbonization of the H_2_ Supply Mix.

To understand the impacts of policies and technology development on the H_2_ sector in plausible future conditions, we develop a range of H_2_ development scenarios from 2025 to 2050 that rely on the socioeconomic projections following the middle-of-the-road, Shared Socioeconomic Pathways (SSP) 2 (39). We vary these scenarios along several key dimensions relevant to H_2_ development, including tax incentives for clean H_2_ production, Bio-H_2_ availability, water electrolysis technology development, and carbon price levels, as shown in Table 1. We first design five supply-side scenarios in the absence of carbon pricing, which vary support levels for H_2_ and emphases on supply technologies: 1) No H_2_: The Baseline scenario assumes no H_2_ supply to end-use sectors and serves as a reference case for quantifying GHG mitigation potential. 2) No policy support for H_2_: The Business-as-usual (BAU) scenario reflects the continuation of existing energy and economic trends without the introduction of new H_2_ or climate policies. Given the limited scale of H_2_ deployment through 2030, this scenario can also approximate the case where the “One Big Beautiful Bill Act” curtails incentives for newly built H_2_ production facilities after 2027. 3) General support for H_2_: The Present scenario incorporates current US IRA 45V tax credits for clean H_2_ production, assuming continued federal support from 2025 to 2050. 4 and 5) Explicit policy support for targeted H_2_ supply technologies: The Water+ and Biomass+ scenarios simulate modified policy environments that promote water electrolysis and biomass gasification technologies, respectively. In addition, given the large uncertainty in the future development of H_2_ supply technologies (14), we consider the sensitivity of varying development trajectories for water electrolysis technology (low, medium, advanced), as well as the availability of Bio-H_2_ technology (with or without Bio-H_2_) for all four scenarios discussed above (BAU, Present, Water +, and Biomass+). Finally, to examine the combined effects of H_2_-specific and economy-wide climate strategies, we combine these supply-side scenarios with four carbon price levels: low, medium, high, and extremely high (Table 2). These economy-wide carbon prices induce changes in both supply and demand sectors, as a higher carbon price makes low-carbon technologies more cost-competitive (40). Details on scenario design are documented in *SI Appendix*, Note 10.

**Table 1.** Scenario setting

Scenario	H_2_ production policy support	Bio-H_2_ availability	Electrolysis development	Carbon price
Baseline	No H_2_ production	No H_2_ production	Low; Medium; Advanced	No carbon price; Low; Medium; High; Extremely high
BAU	No policy support for H_2_	with/without		
Present	General support for H_2_: IRA 45V	with/without		
Water+	Explicit support for H_2_: promoting water electrolysis	with/without		
Biomass+	Explicit support for H_2_: promoting biomass gasification	with/without		

**Table 2.** Carbon price set-up

Year	Low	Medium	High	Extremely high	Unit
2025	16.8	33.6	67.3	67.2	$2020 tonne^−1^ CO_2_
2030	32.6	65.2	130.3	130.3	$2020 tonne^−1^ CO_2_
2035	32.6	65.2	130.3	154.5	$2020 tonne^−1^ CO_2_
2040	32.6	65.2	130.3	178.7	$2020 tonne^−1^ CO_2_
2045	32.6	65.2	130.3	203.0	$2020 tonne^−1^ CO_2_
2050	32.6	65.2	130.3	227.3	$2020 tonne^−1^ CO_2_

**Fig. 2.**
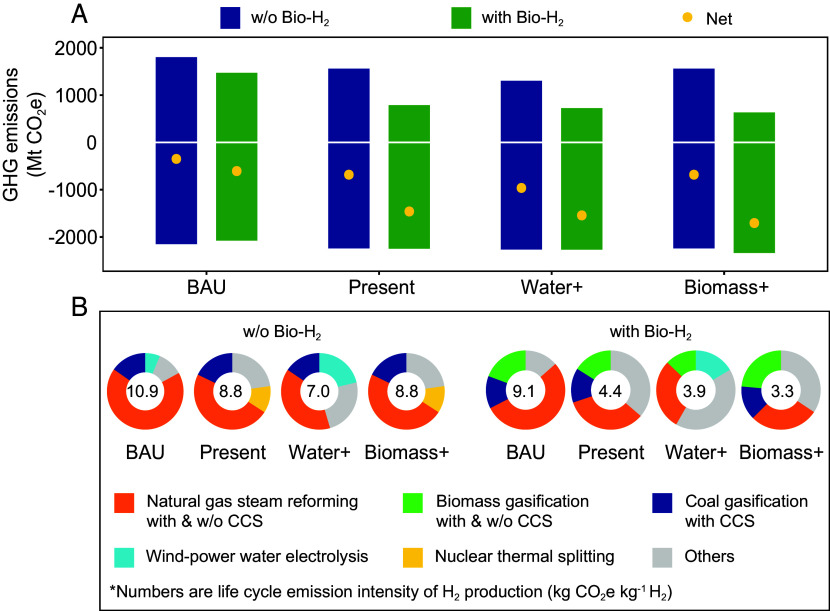
Cumulative GHG emissions and mitigations from 2025 to 2050 across different scenarios and the average life cycle GHG emission intensity of H_2_ with technology breakdown. (*A*) GHG breakdown across different scenarios. The positive emissions are the life cycle emissions from H_2_ production. The negative values show the emission mitigation resulting from the energy displacement of traditional fossil fuels in the end-use sectors. Mt denotes million metric tons. (*B*) H_2_ supply mix breakdown. The top three H_2_ production technologies with the highest market share are shown in the pie chart. The numbers are the H_2_ life cycle GHG emission intensity of each scenario averaged from 2025 to 2050.

**Fig. 3.**
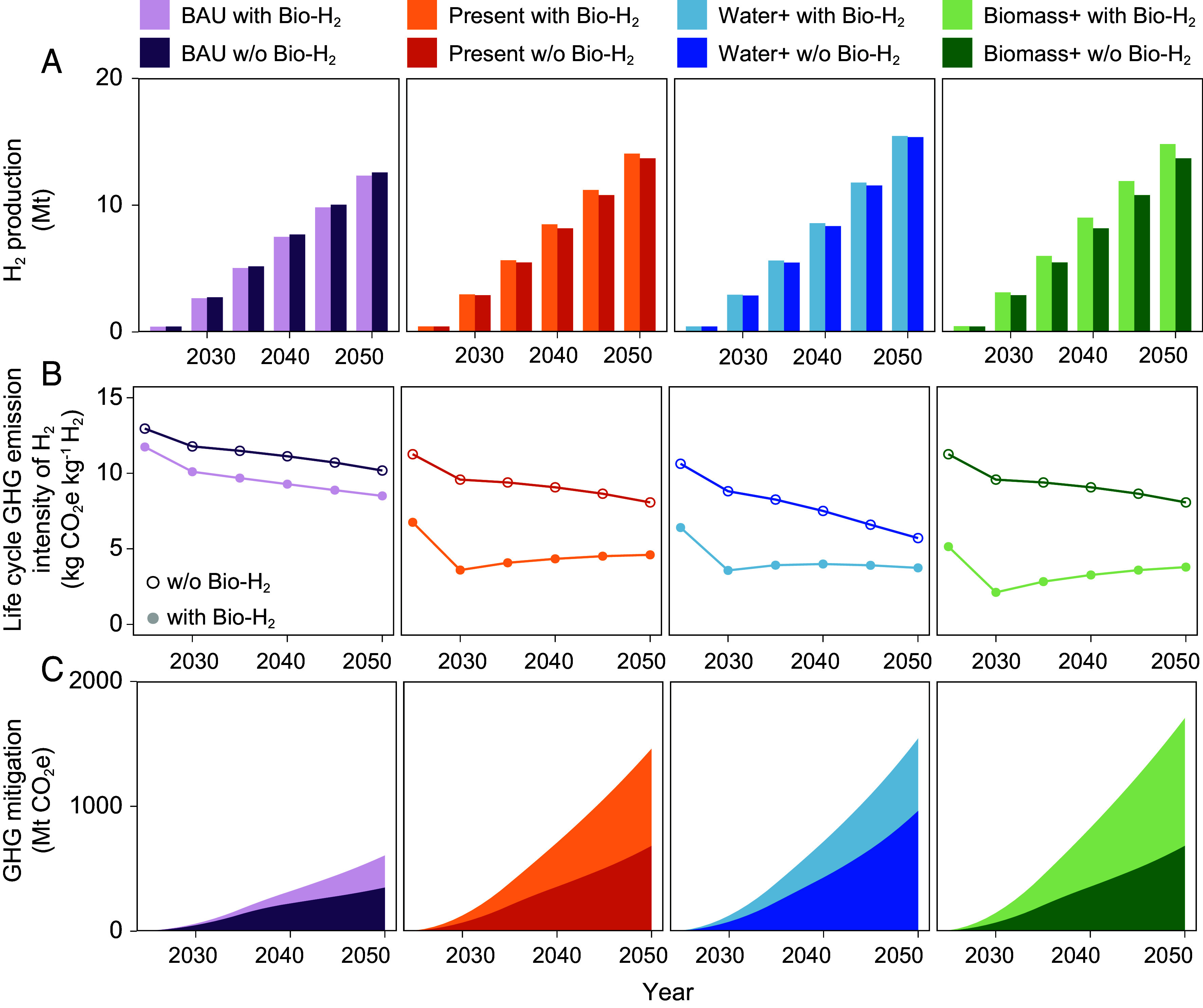
H_2_ production, life cycle GHG emission intensity, and GHG mitigation potential under SSP2 with medium water electrolysis technology development under no carbon price scenario. (*A*) The H_2_ cumulative production from 2025 to 2050 on a 5-y interval in four different scenarios, BAU (purple), Present (orange), Water+ (blue), Biomass+ (green). (*B*) The life cycle GHG emission intensity of H_2_ (dots) across different scenarios for 2025 to 2050 on a 5-y interval. (*C*) The cumulative GHG mitigation potential across four scenarios with different H_2_ production incentives. The GHG emission mitigation is calculated by calculating the emission difference between a given scenario and its counterpart Baseline scenario.

The GHG mitigation potential of H_2_ depends on supply-side GHG emissions and demand-side energy displacement (Fig. 2). Without a carbon price, all scenarios can achieve net negative GHG emissions, indicating greater GHG reduction achieved through energy displacement in H_2_ end-use sectors than the positive GHG emissions from H_2_ production. These net negative emissions are primarily due to the displacement of more carbon-intensive fuels by H_2_ rather than from the direct removal of CO_2_ from the atmosphere. The substantial variations in the net emissions results across different scenarios are mainly driven by differences in life cycle GHG emissions. Specifically, while the demand-side cumulative GHG mitigation is similar across scenarios (~2,000 Mt CO_2_e across all scenarios), the life cycle GHG emissions from H_2_ production vary substantially, ranging from 1,585 Mt CO_2_e in the BAU without Bio-H_2_ scenario to 611 Mt CO_2_e in the Biomass+ with Bio-H_2_ scenario, as shown in Fig. 2*A*. These variations are driven by differences in life cycle GHG emission intensity of H_2_ (Fig. 2*B*) and H_2_ production volume (Fig. 3*A*) across scenarios.

The BAU without Bio-H_2_ scenario exhibits the highest H_2_ life cycle GHG emission intensity (10.9 kg CO_2_e kg^−1^ H_2_), while the Biomass+ with Bio-H_2_ scenario shows the lowest (3.3 kg CO_2_e kg^−1^ H_2_). This disparity is attributed to the different H_2_ supply mixes. The BAU scenario has the highest share of carbon-intensive NG SMR, while the increased share of biomass gasification and biomass gasification combined with carbon capture and storage systems - a form of bioenergy with carbon capture and storage (BECCS) in the Biomass+ scenario results in lower life cycle GHG emission intensity.

### Bio-H_2_–An Essential Transitional Strategy for GHG Mitigation.

Our results show the promising role of Bio-H_2_ in increasing national-level GHG mitigation potential, contributing to additional GHG reductions by substantially lowering H_2_ carbon intensity (Fig. 3*B*), with only a minimal increase of H_2_ production (Fig. 3*A*) in the absence of carbon price. Bio-H_2_ increases the cumulative GHG reduction by 259 Mt, 779 Mt, 615 Mt, and 1071 Mt CO_2_e in BAU, Present, Water+, and Biomass+ scenarios, respectively (Fig. 3*C*). On average, Bio-H_2_ provides 1.6 to 2 times greater GHG reduction potential compared to scenarios without Bio-H_2_. From a temporal perspective, the life cycle GHG emission intensity of H_2_ decreases from 2025 to 2030 due to a shift from NG SMR to Bio-H_2_, particularly BECCS, as the dominant technology. Post-2030, water electrolysis gains more market share as carbon-negative BECCS declines, slightly increasing GHG intensity (Detailed explanation on market expansion and temporal changes in *SI Appendix*, Notes 3 and 4).

Our study focuses on the near-term impact and concludes in 2050, during which biomass gasification remains a cost-competitive technology compared with water electrolysis. Although we assume greater potential for cost reduction for water electrolysis from 2025 to 2050, the production costs of wind-powered electrolysis can only reach parity with biomass gasification around 2045 under the most optimistic scenario (*SI Appendix*, Figs. S1–S3). These production cost estimates include both non-energy components and energy costs related to fuels or electricity. For water electrolysis, the projected cost decline is largely driven by improvements in electrolyzer technology and falling renewable electricity prices. In contrast, the cost reduction of Bio-H_2_ is more limited as the technology is relatively mature with limited cost reduction potential (see the assumptions of cost declines in the *SI Appendix*, Note 8). These differences in cost trajectories shape the modeled H_2_ supply mix and associated mitigation outcomes. Our results highlight the critical role of Bio-H_2_ in enabling near-term GHG reductions under policy scenarios without carbon pricing. By utilizing existing biomass resources, early deployment of Bio-H_2_ supports immediate decarbonization while bridging the transition to lower-cost electrolytic H_2_ in the longer term.

**Fig. 4.**
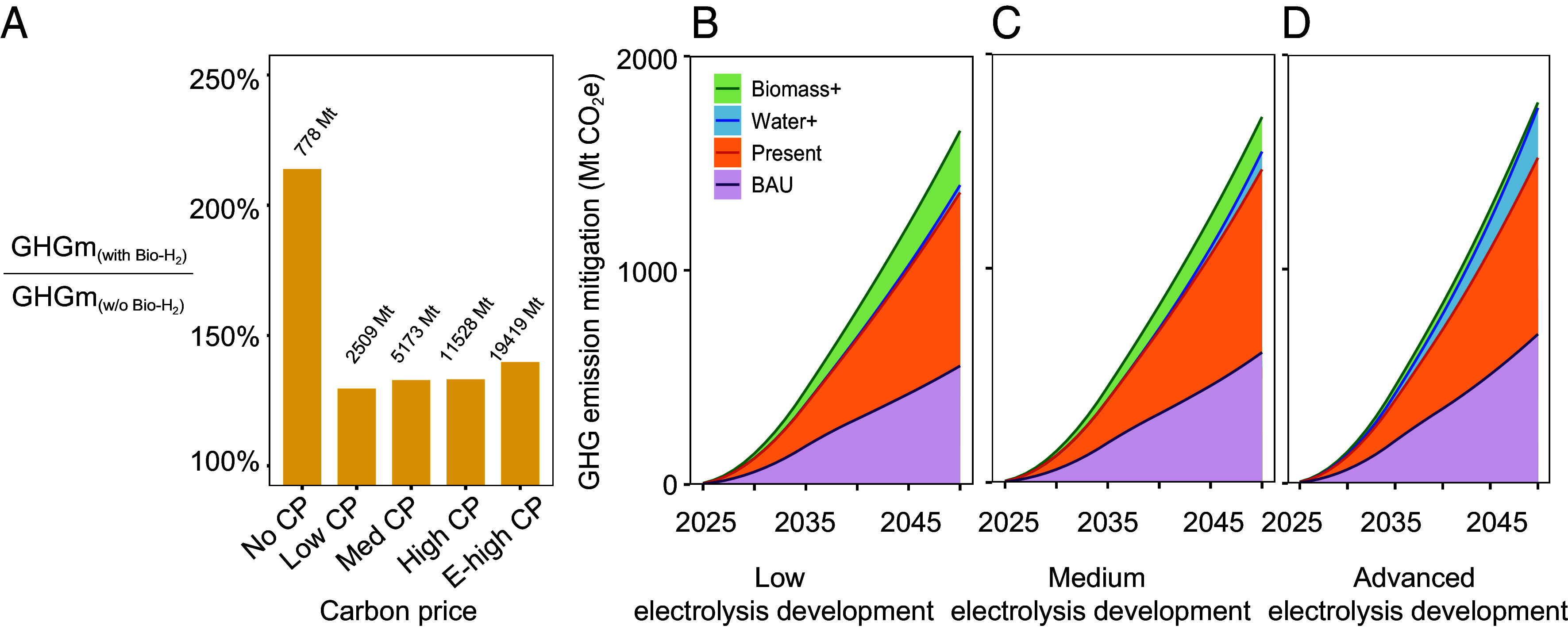
GHG emission mitigation comparison across carbon price and water electrolysis development scenarios. (*A*) GHG mitigation (GHGm) ratios of Bio-H_2_ scenarios to scenarios without Bio-H_2_ under the Present scenario across five carbon price (CP) levels: no CP, low CP, medium CP, high CP, and extremely high CP (E-high CP). The carbon mitigation shown represents the cumulative impact (2025 to 2050), compared to a baseline with no H_2_ and no carbon price. The values of additional GHG mitigation of Bio-H_2_ scenarios compared to those without Bio-H_2_ are shown at the top of the bars. (*B*–*D*) Cumulative GHG emission mitigation potential with no carbon price for BAU, Present, Water+, and Biomass+ scenarios under low, medium, and advanced electrolysis development, respectively.

We also find that higher carbon prices lead to greater additional GHG mitigation from Bio-H_2_, driven by the highly decarbonized H_2_ mix and increased H_2_ production (*SI Appendix*, Figs. S11 and S12). As the carbon price increases from zero to the highest trajectory (i.e., $67 tonne^−1^ CO_2_ in 2025 to $227 tonne^−1^ CO_2_ in 2050), the additional GHG mitigation brought by Bio-H_2_ increases from 778 Mt, 2,509 Mt, 5,173 Mt, 11,528 Mt, and 19,419 Mt CO_2_e under no-, low-, medium-, high-, and extremely high-carbon price trajectories, respectively. However, higher carbon prices lead to smaller relative mitigation advantages for Bio-H_2_ (Fig. 4*A*). For instance, in the present scenario, the GHG mitigation in the Bio-H_2_ scenario is 214% of that in the scenario without Bio-H_2_ when there is no carbon price. This percentage drops to 130%, 133%, 133%, and 140% from low to extremely high carbon price. In addition, the benefits of promoting Bio-H_2_ as a transitional strategy continue diminishing as the carbon price rises. Without a carbon price, the Biomass+ scenario provides 27% more GHG mitigation than the Water+ scenario, but this advantage decreases to 8% and 5% under low and high carbon prices, respectively (*SI Appendix*, Table S1).

We further explore how the tax incentives in the Present scenario influence mitigation potential across scenarios with low, medium, and advanced development of water electrolysis technologies (Fig. 4 *B*–*D*). While much attention has been on water electrolytic H_2_, our findings suggest that promoting Bio-H_2_ offers greater interim GHG mitigation benefits, especially when rapid, large-scale deployment of water electrolysis is not feasible. Under the least developed condition for water electrolysis, promoting Bio-H_2_ (Biomass+ scenario) provides an additional 253 Mt CO_2_e mitigation compared to promoting electrolytic-H_2_ (Water+ scenario). This extra mitigation is around 45.8% of the total GHG mitigation of the BAU scenario. This mitigation advantage of Bio-H_2_ decreases to 25 Mt CO_2_e with advanced water electrolysis technology development (*SI Appendix*, Table S1).

### Demand-Side Decarbonization Misalignment.

On the demand side, we identify a misalignment between H_2_ adoption and its GHG mitigation intensity. We demonstrate this misalignment by first calculating sector-specific GHG mitigation intensity (measured as Mt CO_2_e of GHG reduced per EJ of H_2_ consumed) and ranking the end-use sectors based on their average GHG mitigation intensity. Next, we quantify sectoral H_2_ consumption simulated by the GCAM and rank the sectors based on the aggregate H_2_ adoption level. By comparing these two rankings, we identify sectors that have high GHG mitigation intensity but low H_2_ adoption, which demonstrates missed opportunities.

**Fig. 5.**
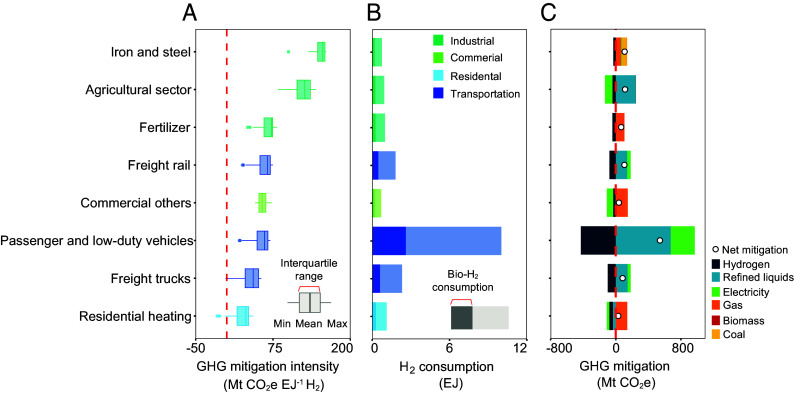
The life cycle GHG mitigation intensity, consumption, and national GHG mitigation of H_2_. (*A*) The life cycle GHG mitigation potential of 1 EJ H_2_ in end-use sectors. The box plots reflect the uncertainty from the 12 scenario settings. Only sectors with H_2_ consumption larger than 3% of the total H_2_ production are presented. The box spans the 25th to 75th percentiles, with the internal line denoting the median. Whiskers extend from the first quartile to the smallest observation within 1.5 times the interquartile range below it, and from the third quartile to the largest observation within 1.5 times the interquartile range above it. (*B*) The sector-wise cumulative H_2_ consumption averaged from all scenarios (2025 to 2050), including the industrial, commercial, residential, and transportation sectors. (*C*) The sector-wise cumulative GHG mitigation breakdown averaged from all scenarios. Bars represent cumulative GHG mitigation by fuel, which is the emission difference from a given fuel between the Baseline scenario and its counterpart scenario with H_2_ supply; positive (or negative) numbers indicate lower (or higher) emissions than the Baseline. Dots are the summation of each fuel breakdown, representing the net cumulative GHG mitigated from 2025 to 2050. Positive values indicate GHG mitigation. Negative values indicate that more GHG emissions result from introducing H_2_ in the sector.

Several industrial sectors (e.g., iron and steel, agricultural, and fertilizer) show high GHG mitigation intensity under no carbon price trajectory (Fig. 5*A*), but their total GHG mitigation remains modest (Fig. 5*C*) due to low H_2_ consumption (Fig. 5*B*). For instance, the average mitigation potential per unit H_2_ use is approximately 147 Mt CO_2_e EJ^−1^ for the iron and steel sector and 54 Mt CO_2_e EJ^−1^ for passenger and light-duty vehicles, while their average H_2_ consumption is 0.8 EJ and 10 EJ, respectively. These misalignments reveal that high-GHG mitigation end-use sectors are not fully leveraging H_2_’s potential, which are missed opportunities. In contrast, the transportation sector, which has the largest H_2_ consumption (75% of total H_2_ consumption), exhibits lower mitigation intensity. The high H_2_ consumption in the transportation sector is not surprising, given existing US policies that support alternative fuels, including H_2_ (41–44). This result is also consistent with previous IAM studies (45, 46). The sectoral differences in mitigation intensity arise from the types of fuels displaced by H_2_ use (Fig. 5*C*), which vary in carbon intensity. For instance, H_2_ used in the iron and steel sector displaces a larger share of coal, leading to high emissions reductions per EJ of H_2_. In contrast, in commercial and residential buildings, H_2_ mainly replaces natural gas, which has a lower carbon intensity and results in smaller mitigation per EJ of H_2_.

In the absence of carbon prices, low H_2_ consumption in industrial sectors is attributed to the cost competitiveness of conventional energy and technology supplies. For instance, in the iron and steel sector, traditional steel-making technologies, e.g., blast furnace-basic oxygen furnace, are more cost-competitive currently than electric arc furnace direct reduction with H_2_ (*SI Appendix*, Table S2). The degree to which conventional technologies are displaced by H_2_ depends on the relative costs of these technologies (modeled with the cost components of the GCAM choice functions) as well as how quickly new technologies can penetrate the market given the incumbents’ advantages (47, 48) (modeled with the non-cost component of the choice function). Details of the choice functions are documented in *SI Appendix*, Note 9. Sectors where H_2_ outcompetes other alternatives receive higher deployment rates. Conversely, in sectors where H_2_ remains less competitive than other technologies, its adoption is constrained.

To explore the effects of broader climate policies and economy-wide decarbonization on the demand side, we further analyze the impact of carbon prices on the misalignment. At low and medium carbon prices, the misalignment persists. For example, the agriculture and iron and steel sectors continue to have the highest GHG mitigation intensity but rank low in H_2_ consumption (*SI Appendix*, Figs. S4–S7*A*). As the carbon price increases, the extent of the misalignment decreases, due to diminishing differences in the mitigation intensity across sectors (*SI Appendix*, Figs. S4–S7*B*) and more balanced H_2_ consumption (*SI Appendix*, Fig. S8). This is driven by the shrinking cost differences between H_2_ and conventional technologies, when fossil technologies become more expensive due to a high price on carbon. Quantitatively, the H_2_ consumption in the iron and steel sector increases significantly from 0.8 EJ without a carbon price to 12.9 EJ under the highest carbon price, followed by an increase of 5.3 EJ and 4.4 EJ in the cement and fertilizer sectors, respectively. Under the extremely high carbon price, the iron and steel sector achieves the second-largest GHG mitigation at 2,626 Mt CO_2_e, followed by the fertilizer (1,457 Mt CO_2_e) and cement (1,200 Mt CO_2_e) sectors (*SI Appendix*, Figs. S4–S7*C*). The larger increase in H_2_ consumption in the industrial sector is attributed to the higher share of energy-related costs in total H_2_ production costs (e.g., ~45% in iron and steel, ~50% in fertilizer) compared to transportation sectors (e.g., ~20% in passenger and low-duty vehicles) (*SI Appendix*, Fig. S9). Since carbon pricing primarily affects energy-related emissions, the cost penalties for carbon-intensive technologies in industrial sectors are higher. This, in turn, favors the adoption of less carbon-intensive H_2_ technologies in industrial sectors.

## Discussion

H_2_’s versatility as an energy carrier offers promising opportunities in nontraditional applications, particularly in sectors where low-carbon alternatives are limited. Realizing its climate benefits depends not only on the decarbonization of H_2_ supply mix but also on the strategic deployment of H_2_ in end-use applications. Looking ahead to 2050, mitigation efforts in the next 25 y are crucial to ensure the ambition of reaching net-zero emissions becomes a reality (49, 50). Our results suggest that, in the long term, advances in water electrolysis technology could decarbonize the H_2_ production mix on the supply side, and the stringent carbon price could help address the misalignment on the demand side. However, such developments in technology and policy are unlikely to materialize in the short term, given the current political and market landscape in the United States, where water electrolysis technology faces scale-up barriers (14, 51) and recent federal policy reversal is expected to slow down clean energy investments and the country’s overall climate policy ambition (52). Therefore, short-term solutions become critical. Accordingly, our study provides insights into how mature technologies such as Bio-H_2_ and targeted H_2_ policies can achieve climate benefits before long-term developments are realized.

On the supply side, we highlight the critical role of Bio-H_2_ in short-term GHG mitigation under less H_2_-favorable situations, such as without clean H_2_ production tax incentives, low water electrolysis development, or without carbon pricing. These H_2_-unfavorable situations are highly probable due to the recent approval of the One Big Beautiful Bill Act. Under each of these conditions, Bio-H_2_ significantly decreases the life cycle GHG emission intensity of the H_2_ supply mix and substantially increases total GHG mitigation compared to scenarios without Bio-H_2_ but with clean H_2_ production incentives, advanced water electrolysis, or national-level carbon pricing, respectively. Additionally, the sole carbon-negative production pathway, BECCS H_2_, has the potential to scale given the tremendous interest and growth of the voluntary and compliance carbon market (53). To fully leverage its potential, targeted incentives are needed to lower the current high cost of BECCS H_2_ (16, 54) and facilitate its market entry. To decrease the carbon intensity of the H_2_ supply mix, Bio-H_2_ can serve as a critical transitional technology for clean H_2_ supply until the costs of water electrolysis can be substantially reduced. Gasification is also a versatile technology that can be adapted to produce value-added fuels and chemicals beyond H_2_, laying the foundation for the future low-carbon transition of various industrial sectors.

The US H_2_ policy has long been focusing on decarbonizing the H_2_ supply side by replacing conventional production technologies with cleaner alternatives, represented by the IRA 45V tax credit (6, 55). In contrast, relatively little attention has been paid to stimulating the demand-side deployment. This supply-side emphasis on setting domestic H_2_ production goals is also common in other regions, including the European Union’s REPowerEU plan (56), Japan’s Basic Hydrogen Strategy (57), and Australia’s National Hydrogen Strategy (58). Our study underscores the importance of demand-side solutions. We identify a misalignment between where H_2_ is used and where it can offer the higher mitigation intensity in the absence of a national-level carbon price. As a national carbon pricing scheme is unlikely to be fully implemented in the near term in the United States, targeted policy support remains essential (59). Prioritizing H_2_ deployment and in sectors with a greater scale of mitigation potential can improve the overall climate benefits of H_2_ strategies. In addition, while sectors with large H_2_ demand may deliver significant total emissions reductions, those with high mitigation intensity per unit of H_2_ represent missed opportunities. Recognizing both dimensions, scale and intensity, is critical to realizing H_2_’s full decarbonization potential. These insights can not only inform US policy but also guide other countries facing similar supply-heavy strategies and limited demand-side actions.

Effective demand-side solutions are twofold. First, increasing GHG mitigation intensity by advancing H_2_ technology at the process level in the industrial sectors, with particular attention to Bio-H_2_ coproduction and utilization. Coproduction refers to integrated systems that generate H_2_ alongside other biobased products such as biofuels or biochemicals, while utilization emphasizes the direct use of Bio-H_2_ in industrial processes, such as high-temperature heat or chemical feedstock (60, 61). For instance, in the iron and steel sector, further technical advancements are required to scale up bio-syngas facilities for direct H_2_ use in steelmaking. This adoption will need careful consideration on whether to construct new plants or retrofit existing ones on a case-by-case basis (62). Second, expanding H_2_ consumption in the industrial sector by appropriate market instruments. Direct subsidies or other incentives aimed at lowering the cost of H_2_ adoption in industry could accelerate H_2_ deployment. Our results show that a national carbon price applied across all sectors leads to a greater increase in H_2_ use in the industry sectors compared to transportation, largely due to the higher share of energy costs in energy-intensive industrial sectors. This cost structure makes these industrial sectors particularly sensitive to energy-related policy interventions. While broad carbon pricing can drive H_2_ adoption, targeted subsidies or incentives specifically designed to lower the cost of H_2_ adoption in industry could be even more effective in accelerating deployment and achieving deeper emissions reductions.

This study has several limitations. Our analysis focuses on H_2_ as an energy source, excluding H_2_ used as feedstock, as the latter is not included in the GCAM calibration (63). Since H_2_ use as feedstock in chemicals and refineries has been intensively studied in the literature (30, 33, 64), our study complements previous research by providing valuable insights into the underexplored use of H_2_ as an energy carrier. Additionally, subnational differences of the US H_2_ sectors are not investigated due to the lack of region-specific data and modeling capability. Future research can explore the state-level H_2_ sector using our modeling framework when subnational data becomes available. Due to the computational complexity of LCA-IAM models, we do not address all parametric and structural uncertainties but perform sensitivity analysis to identify key factors affecting life cycle emissions (*SI Appendix*, Fig. S10 and Note 6). We also do not account for H_2_ leakage and associated global warming impact during the transmission and distribution of centrally produced H_2_, given its minimal impacts on life cycle emissions according to previous studies (65–67) (*SI Appendix*, Note 7).

## Materials and Methods

### Prospective LCA.

We conduct a prospective, consequential LCA to assess the environmental impacts of H_2_ production and applications. The system boundary includes production, transmission and distribution, and end-use combustion. Life cycle inventory (LCI) data were derived from the GHGs, Regulated Emissions, and Energy use in Technologies (GREET) model (68). We use a consequential approach to consider changes in H_2_ supply mix and applications in various end-use sectors in the future, leveraging GCAM. All GHGs are quantified using global warming potential (GWP)-100-y, following Intergovernmental Panel on Climate Change (IPCC) guidelines (2).

The life cycle GHG emission intensity of H_2_ is estimated using the Eq. **1**.

[1] $$\begin{array}{c} {\mathrm{EI}}_{i,t}={\mathrm{GHGI}}_{production,i,t}+{\mathrm{GHGI}}_{transport,i,t}+{\mathrm{GHGI}}_{enduse,i,t} \end{array}.$$

${\mathrm{EI}}_{i,t}$ is the life cycle GHG emission intensity (Mt CO_2_e EJ^−1^) of H_2_ technology i in year *t*. i includes technologies such as biomass gasification, natural gas SMR, water electrolysis, etc. The full list of technologies is in *SI Appendix*, Table S10. ${\mathrm{GHGI}}_{production,i,t}$, ${\mathrm{GHGI}}_{production,i,t}$, and ${\mathrm{GHGI}}_{production,i,t}$, are the GHG emission intensities of 1 EJ of H_2_ (Mt CO_2_e EJ^−1^) in year *t* from the production, transportation, and end-use stages, respectively.

The GHG emission intensities of H_2_ production (${\mathrm{GHGI}}_{production,i,t}$) are estimated using the following Eq. **2**.

[2] $$\begin{array}{c} {\mathrm{GHGI}}_{{\mathrm{production}}_{i,t}}=\sum_{q}{\mathrm{GHGI}}_{i.q}*\left(\sum_{2025}^{t}\left(P_{i,t}*C_{i,q,t}\right)\right). \end{array}$$

${\mathrm{GHGI}}_{i.q}$ is the life cycle GHG emission intensities (Mt CO_2_e EJ^−1^) of feedstock *q* used in H_2_ production technology *i*. These emission intensities include both direct and upstream emissions for H_2_ production. Direct emissions refer to emissions generated at the point of H_2_ production, such as the combustion of natural gas, which results in CO_2_ and CH_4_ emissions. Indirect emissions arise from the upstream stages of fuel and material supply chains—extraction, processing, and transportation of energy feedstocks such as coal, refined oil, natural gas. $P_{i,t}$ is the percentage of H_2_ produced from technology *i* in year *t*, relative to the total H_2_ produced in year *t*. A summation is performed from year 2025 to year *t* as H_2_ produced in year *t* consists of H_2_ produced from plants built in different years. The earliest year is 2025. H_2_ production from plants built prior to 2025 and used as fuels is close to 0. $C_{i,q,t}$ is the input–output coefficient of converting feedstock *q* (input in EJ) into H_2_ (output in EJ). This coefficient varies by year to represent the efficiency improvement. The full list of $C_{i,q,t}$ in *SI Appendix*, Table S4.

The GHG emissions associated with fuels displaced by H_2_ are estimated by the Eq. **3**.

[3] $$\begin{array}{c} {\mathrm{EI}}_{f,t}=\sum_{n}{\mathrm{GHGI}}_{production,f,n,t}*P_{f,n,t}+{\mathrm{GHGI}}_{transport,f,t}+{\mathrm{GHGI}}_{enduse,f,t} \end{array}.$$

${\mathrm{EI}}_{f,t}$ is the life cycle emission intensity (Mt CO_2_e EJ^−1^) of a displaced fuel *f* in year *t*. ${\mathrm{GHGI}}_{production,f,n,t}$ is the production-stage GHG emission intensities (Mt CO_2_e EJ^−1^) of a displaced fuel *f* produced from technology *n* in year *t*. *f* includes electricity, natural gas, etc. A full list is in *SI Appendix*, Table S5. *n* represents production technology pathways for each *f*. For example, for electricity, the *n* includes electricity production pathways. The full list is in *SI Appendix*, Table S3. ${\mathrm{GHGI}}_{production,f,t}$, and ${\mathrm{GHGI}}_{production,f,t}$, are the GHG emission intensities (Mt CO_2_e EJ^−1^) of 1 EJ of displaced fuel *f* in year *t* from transportation and end-use stages, respectively from GREET. $P_{f,n,t}$ is the percentage of fuel *f* produced from technology *n* in year *t*, relative to the total *f* produced in year *t*.

The Eq. **4** calculates the cumulative GHG mitigation (${\mathrm{GHG}}_{\mathrm{mitigation}}$, Mt CO_2_e), considering both yearly emissions from the production stage as the supply-side (${\mathrm{GHG}}_{supply,t}$, Mt CO_2_e) and the yearly emission mitigation from energy substitution from the end-use sectors as the demand-side GHG mitigation (${\mathrm{GHG}}_{demand,t}$, Mt CO_2_e), applicable to all scenarios.

[4] $$\begin{array}{c} {\mathrm{GHG}}_{\mathrm{mitigation}}\;=\sum_{t=2025}^{2050}\left(GHG_{demand,t}-{\mathrm{GHG}}_{supply,t}\right) \end{array}.$$

The Eq. **5** calculates the total life cycle GHG emissions of H_2_ production in each year considering different technologies and H_2_ production volume. $S_{t}$ is the H_2_ supply (EJ) in the year *t*. $m_{i,t}$ is the percent of an H_2_ production technology *i* in year *t*.

[5] $$\begin{array}{c} {\mathrm{GHG}}_{supply,t\;=}\sum_{i}S_{t}*m_{i,t}*EI_{i,t} \end{array}.$$

The demand-side GHG mitigation is estimated using the Eq. **6** where $D_{f,j,t}$ is the consumption (EJ) of displaced fuel *f* in sector *j* in the year *t*.

[6] $$\begin{array}{c} {\mathrm{GHG}}_{demand,t\;=}\sum_{f,j}\left({\left(D_{f,j,t}*EI_{f,t}\right)}_{Baseline\;scenario}-{\left(D_{f,j,t}*EI_{f,t}\right)}_{\mathrm{Incentive}\;\mathrm{scenario}}\right) \end{array}.$$

### IAM.

Our scenarios are developed using GCAM v7.0. By incorporating various input assumptions related to socioeconomic drivers, technology costs, and policy actions, GCAM projects future patterns at 5-y intervals within a partial equilibrium economic modeling framework (63). GCAM includes 32 geoeconomic economic regions with the United States being a standalone modeling region. In this study, we change the technology and policy assumptions only for the United States, while keeping the assumptions for the rest of the regions the same as the default assumptions in the default case. Since GCAM is an open-source, open-data model with a detailed online documentation (https://jgcri.github.io/gcam-doc/v7.0/overview.html), below we only provide relevant model descriptions related to H_2_ representations that are particularly relevant to this study.

On the supply side, GCAM includes both centralized and decentralized H_2_ production. We modify GCAM to include additional on-site H_2_ production technologies, including electrolysis powered by solar and wind energy (48), natural gas with CCS, and biomass gasification. These on-site technologies have gained increasing interest in recent years (69, 70). Assumptions for on-site renewable electrolysis and natural gas with CCS are based on existing literature (48). The cost assumption of on-site biomass gasification is interpolated from the H2A model (20) and the efficiency assumption remains the same as the central biomass gasification process. Further details regarding GCAM modifications are shown in *SI Appendix*, Note 1.

On the demand side, H_2_ is adopted across the industry, transport, and building sectors in GCAM. The adoption of H_2_ is simulated by a choice model driven by cost-based competition and market preferences, with assumptions that evolve over time to reflect market maturity and infrastructure development. This approach accounts for region-specific preferences (such as social or cultural factors) and enables a more realistic simulation of new technology adoption. The choice model functions and details are shown in the *SI Appendix*, Note 9. In industrial sectors, H_2_ is used for process heat and cogeneration. In the transport sector, H_2_-powered technologies (e.g., fuel cell vehicles) are modeled for passenger vehicles, buses, freight trucks, marine vessels, and freight rail. In the buildings sector, H_2_ is used to provide district heating, direct residential and commercial heating, and other energy services such as water heating and cooking (48). Refueling infrastructure and storage are accounted for in the transportation sector, with assumptions drawn from the Hydrogen Delivery Scenario Analysis Model (71).

### Soft Link between Models.

The GREET model is used for LCA calculations, providing detailed LCI data for all H_2_ production pathways considered. The GCAM is used to provide fuel production and consumption that inform scenario-specific H_2_ supply and demand mixes. We select GREET because it is tailored to the US context and provides detailed process-level LCI that we use to estimate carbon intensity with greater granularity than the carbon-balance approach employed by GCAM. To ensure consistency between the two models, we first align secondary energy carriers such as electricity and H_2_ by incorporating input–output coefficients from GCAM into GREET. These coefficients represent energy conversion efficiency from primary to secondary fuel, e.g., the efficiency of electricity generation from fossil or renewable sources (*SI Appendix*, Table S4). Second, to further harmonize the models, we adjust GREET’s background electricity emission factors to reflect GCAM’s evolving electricity supply mix across scenarios. This step is particularly important for prospective LCA studies, where electricity emissions are often a key source of uncertainty. Instead of relying solely on GREET’s default electricity emission factors with default mixes, we extract the electricity generation mix from GCAM under each carbon price scenario and recalculate the corresponding emission factor within GREET (expressed in g CO_2_e per kWh of electricity). Our emissions calculations are conducted independently from those of GCAM. We do not use GCAM’s internal emission output, given its lack of inclusion of process-based LCI; instead, we rely on GCAM for energy flow and market data while performing standalone emissions analysis using GREET.

## Supplementary Material

Appendix 01 (PDF)

## Data Availability

The necessary inputs and outputs for tables, data, and figures in this study that support this research are available in the article and/or *SI Appendix*. The GCAM model is publicly available (47). The modification and the scenario files used in this study are accessible for download (72).
