# Supplementary material for: Supply–demand strategies for near-term climate benefits from hydrogen in the United States
Source: Proc Natl Acad Sci U S A. 2025 Oct 6;122(41):e2519606122. doi: 10.1073/pnas.2519606122 (PMC12541446; doi:10.1073/pnas.2519606122)
Supplement: Supplementary file 1 — Appendix 01 (PDF) [file pnas.2519606122.sapp.pdf]

PNAS

Supporting Information for
Supply-demand strategies for near-term climate benefits from hydrogen in the United States

Youyi Xu[a], Wei Peng[b,c], Yuan Yao[a,d*]

[a]Center for Industrial Ecology, Yale School of the Environment, Yale University, New Haven, CT 06511, USA

[b]Andlinger Center for Energy and the Environment, Princeton University, Princeton, NJ, USA

[c]School of Public and International Affairs, Princeton University, Princeton, NJ, USA

[d]Chemical and Environmental Engineering, Yale School of Engineering and Applied Science, Yale University, New Haven, CT, USA

* Yuan Yao

Email: y.yao@yale.edu

This PDF file includes:

Supporting Notes 1-10
Figures S1 to S12
Tables S1 to S13
SI References

Contents

Supplementary Notes 1

Supplementary Note 1: GCAM modifications 1
Supplementary Note 2: GHG intensities of electricity and refined liquids 2
Supplementary Note 3: H_2 market expansion 5
Supplementary Note 4: H_2 supply mix temporal changes 6
Supplementary Note 5: H_2 consumption cap 7
Supplementary Note 6: LCA sensitivity analysis 8
Supplementary Note 7: H_2 leakage and losses during transport 9
Supplementary Note 8: H_2 production cost assumptions 10
Supplementary Note 9: GCAM choice model 11
Supplementary Note 10: Scenario setting 12

SI Figures 13

Fig. S1. H_2 central production prices under BAU scenario. 13
Fig. S2. H_2 onsite production prices under BAU scenario. 14
Fig. S3. H_2 onsite (forecourt) production prices under BAU scenario. 15
Fig. S4. Sectoral H_2 consumption and mitigation potential mismatch (low carbon price scenario). 16
Fig. S5. Sectoral H_2 consumption and mitigation potential mismatch (medium carbon price scenario). 17
Fig. S6. Sectoral H_2 consumption and mitigation potential mismatch (high carbon price scenario). 18
Fig. S7. Sectoral H_2 consumption and mitigation potential mismatch (extremely high carbon price scenario). 19
Fig. S8. Variances across different carbon price scenarios. 20
Fig. S9. Non-energy costs percent across transportation and industrial sectors. 21
Fig. S10. Sensitivity analysis of the life cycle emission for each H_2 production pathway. 22
Fig. S11. H_2 production under different carbon price trajectories. 23
Fig. S12. H_2 production mix intensity under different carbon price trajectories. 24

SI Tables 25

Table S1. Results of cumulative GHG emission mitigation compared with no H_2 and no carbon price scenarios with medium water electrolysis development 25
Table S2. Results of costs of various steelmaking technologies under the Present scenario with medium water electrolysis technology development in GCAM 26
Table S3. Results of electricity technology baseline emission factors in 2020 27
Table S4. Feedstock input-output coefficients of H_2 production in GCAM assumption 28
Table S5. Results of primary fuels emission factors in Present 45V scenario with medium water electrolysis development and with Bio-H_2 29
Table S6. H_2 Tax incentives set up (unit: \$1975 GJ^{-1} H_2) 30
Table S7. Breakdown of 2022 US refiner and blender net production of traditional oil refined products 31
Table S8. H_2 consumption cap 32
Table S9. Water electrolysis cost assumptions in GCAM (unit: \$1975 GJ^{-1} electricity) 33
Table S10. Life cycle GHG emission intensity of individual H_2 production pathways in each year (unit: Mt CO_2e EJ^{-1} H_2) 34
Table S11. Electricity scale factor and GHG emission intensity 37
Table S12. H_2 production percent yearly breakdown under no carbon price scenario 42
Table S13. IRA 45V tax incentives for clean H_2 production 47

SI References 48

Supplementary Notes

Supplementary Note 1: GCAM modifications

In Global Change Analysis Model (GCAM), H_2 can be produced via two approaches. One is centralized production at large-scale facilities, delivering H_2 to end users. The other is de-centralized production at the end user's location. Centralized technologies include NG SMR, NG SMR with CCS, coal gasification, coal gasification with CCS, grid-power electrolysis, wind-powered water electrolysis, solar-powered water electrolysis, biomass gasification, and BECCS. Fewer technologies are included in the decentralized production, only grid-powered electrolysis and NG SMR. First, we apply the modifications of onsite hydrogen (H_2) production to the base GCAM v7.0 from the existing literature (1) to include solar- and wind-water electrolysis onsite production. The default GCAM v7.0 includes H_2 central production from biomass gasification, biomass gasification with CCS, coal gasification, grid-electricity electrolysis, natural gas steam reforming, natural gas steam reforming with CCS, nuclear thermal splitting, solar-electricity electrolysis, wind-electricity electrolysis. For onsite production, grid-electrolysis and natural gas steam reforming are included. Second, we add the onsite biomass gasification H_2 production pathway to explore a more comprehensive set of hydrogen applications across key end-use sectors. The non-energy costs of onsite biomass gasification are interpolated from H2A model (2) which has detailed cost data for central and distributed H_2 production pathways. The H_2 distribution costs are derived from Argonne's Hydrogen Delivery Scenario Analysis Model (HDSAM) (3). The GCAM shareweight setting, water costs, and biomass feedstock conversion efficiency for on-site biomass gasification are the same as the central biomass gasification. The onsite biomass gasification with CCS is not considered in our analysis due to the high costs for a small-scale plant to include CCS technology.

Supplementary Note 2: GHG intensities of electricity and refined liquids

This note documents the detailed calculation of greenhouse gas (GHG) intensities for two energy fuels—electricity and refined liquids—used in hydrogen production. The calculation requires careful consideration to ensure that the GHG intensities are properly weighted across various production pathways in a given GCAM scenario.

We use The Greenhouse Gases, Regulated Emissions, and Energy Use in Transportation (GREET) model to calculate the yearly upstream emission intensities and combustion emissions of H_2 and displaced fuels (electricity, natural gas, refined liquids, coal, biomass). The input-output coefficients represent the energy transformation efficiency from the primary fuel (input) to the secondary fuel (output). For the biomass gasification pathway, we choose poplar as the representative biomass feedstock to align the cost assumption with the H2A model (2) which is also the cost assumption used in GCAM. H_2 transportation from central production facilities to end users is assumed to be 100 km, except for central H_2 production from water electrolysis, which is assumed to require 500 km of transmission. The increased transport distance builds on the assumption that electrolyzers will be co-located with wind or solar installations, which are generally far from the H_2 demand centers (1). In the end-use stage, H_2 combustion does not generate GHGs, we mainly include GHGs generated by combusting displaced fuels.

In the electricity sector, for fossil-based and biomass-based power plants, first, we apply the input-output coefficients, defined in equation (S1), of various power plants in GCAM to GREET to avoid any mismatches. The year 2020 is chosen as the baseline, the baseline emission factors are shown in **Table S3**. We derive the emission factor of electricity from the GREET model in CO_2e mmBtu^{-1} electricity generated from all kinds of power plants in GCAM. **Table S3** documents the input-output coefficient (IO) using the equation (S1).

$$IO_{n,t} = \frac{input}{output} \tag{S1}$$

where $IO_{n,t}$ is the input-output coefficient of electricity production technology n in year t. The $input$ (mmBtu) refers to the major fuel used to produce electricity. For example, coal is the major fuel input for the coal−integrated gasification combined cycle process to produce electricity. The $output$ is the electricity produced (mmBtu). $IO_{n,t}$ is the input-output coefficient of electricity production technology n in year t.

As the input-output coefficients change across years, we then apply a scale factor based on the IO coefficients for 2015, 2025, 2030, 2035, 2040, 2045, and 2050 to calculate the changing electricity GHG intensity. Note that there are multiple inputs for a specific type of power plant. The IO coefficient used to calculate the scale factor is the representative feedstock of the power plant based on the plant's type. For instance, natural gas is the representative feedstock for a natural

gas power plant, and coal is the representative feedstock for a coal-based power plant. Power plants in the past will continue to generate electricity, but their efficiency will not change. For example, the electricity generated in 2025 includes electricity generated from plants both built in 2025 and prior to 2025. We also capture this trend by using the vintage tab in GCAM.

$$u_{n,t} = \frac{IO_{n,t}}{IO_{n,baseline}} \qquad (S2)$$

$$GHGI_{production,elec,n,t} = \sum_{2015}^{t} GHGI_{n,t} * S_{n,t} * u_{n,t} \qquad (S3)$$

where $u_{n,t}$ is the scale factor; $IO_{n,t}$ is the input-output coefficient of technology n in year t; $IO_{n,baseline}$ is the input-output coefficient in year 2020; $GHGI_{n,t}$ is the life cycle GHG intensity (g CO_2e $mmBtu^{-1}$) of electricity production technology n in year t; $GHGI_{n,baseline}$ is the life cycle GHG intensity (g CO_2e $mmBtu^{-1}$) of electricity production technology n in year 2020. As the electricity produced in year t also includes electricity plants built in year $t-1$ with a different EI, so a weighted calculation is performed in equation (S3). As power plants generating electricity in 2025 include those operating since 2015, the calculation traces back to the earliest year, 2015.

Combined with the supply mix of different power plants for electricity generation in GCAM, the electricity emission factor for a given scenario can be determined. The yearly scale factors are provided in **Table S11**. For renewable power plants—solar, wind, geothermal, hydro, and nuclear—we directly extract yearly data from GREET (4).

In the refined liquid sector, the refined liquid acts both as the fuel flows into end-use sectors and the sector that consumes H_2. When considered as the end-use sector of H_2, the GHG emissions in a certain year t, GHG_t(g CO_2e $mmBtu^{-1}$) are calculated as:

$$GHG_t = \sum EI_{f,t} * I_{f,t} - \sum EF_{r,t} * O_{r,t} - GHG_{CCS,t} \qquad (S4)$$

where $EI_{f,t}$ is life cycle emission intensity (g CO_2e $mmBtu^{-1}$) of fuel f in year t; $I_{f,t}$ is the fuel consumption (mmBtu) of fuel f in year t; $EI_{r,t}$ is the life cycle emission intensity (g CO_2e $mmBtu^{-1}$) of a given refined liquid product r in year t ; $O_{r,t}$ is the final output products (mmBtu) of refined liquids from production technology r in year t; $GHG_{CCS,t}$ represents the GHG emissions (g CO_2e) captured by CCS in year t.

In GCAM, Refined liquids production technologies include biomass to liquids, Fischer-Tropsch biofuel, Fischer-Tropsch biofuel with CCS, biodiesel, cellulosic ethanol, cellulosic ethanol with CCS, corn ethanol, coal to liquids, gas to liquids, and traditional oil refining. For the first nine refined liquids production pathways, we derive their refined liquids product life cycle emission factors from GREET (4). As there is no detailed breakdown for the traditional oil refined products, we apply the

U.S. refiner and blender net production of petroleum products in 2022 as proxy[5] to calculate the average $EI_{r,t}$ of the traditional oil refining technology. Only liquid products are considered, namely gasoline, diesel, conventional jet fuel, and natural gas liquids. The volume percentages are transformed into energy percentages to calculate the weighted traditional oil refined products (**Table S7**).

When considered as the fuel for other end-use sectors, the weighted emission intensity of refined liquids:

$$GHGI_{production,refined,n,t} = \frac{\sum_f EI_{f,t} * I_{f,t} - GHG_{CCS,t}}{O} \qquad (S5)$$

where $EI_{f,t}$ is life cycle emission intensity (g CO_2e mmBtu^{-1}) of fuel f in year t; $I_{f,t}$ is the fuel consumption (mmBtu) of fuel f in year t; O is the final output (mmBtu) of refined liquids; GHG_{CCS} represents the GHG emissions (g CO_2e) captured by CCS in year t.

Supplementary Note 3: H_2 market expansion

Our results in the main text **Fig. 2A** show that the inclusion of Bio-H_2 slightly expands the H_2 market, except under the BAU scenario. This is because the cost of biomass gasification is relatively low compared to other H_2 production pathways. Therefore, its inclusion reduces the overall price of the H_2 supply mix, simulating more H_2 consumption. The availability of Bio-H_2 has the most significant impact on the H_2 market size in the Biomass+ scenario. More aggressive tax incentives towards biomass gasification further lower the price of the H_2 supply mix compared with other scenarios, leading to the largest market expansion. For example, the H_2 production quantity expands 0.13 EJ in 2050 after including Bio-H_2 in Biomass+. This compares to the expansion of 0.04 EJ in Present and 0.01 EJ in Water+. On the contrary, in the BAU scenario, the inclusion of Bio-H_2 acquiring parts of the market share dominated by the cheaper natural gas steam methane reforming (SMR) H_2, resulting in a higher supply mix cost, which further leads to a diminished H_2 market. The yearly increased H_2 consumption is attributed to a more accessible transmission and distribution system and higher acceptance in the end-use sectors due to cost competitiveness of H_2 compared with other fuels fulfilling the same service.

Supplementary Note 4: H_2 supply mix temporal changes

From a temporal perspective, the life cycle GHG intensity of H_2 decreases from 2025 to 2030 and increases from 2030 in scenarios with both Bio-H_2 supply and tax credits. NG SMR H_2 is the dominant technology in 2025, resulting in high life cycle GHG emission intensity in that year. As the market share of NG SMR decreases and the share of Bio-H_2, particularly those from bioenergy with carbon capture and storage systems (BECCS), increases, the life cycle GHG emission intensity is reduced. Biomass gasification reaches the highest market share in 2030 (**Table S12**) due to cost competitiveness (**Fig. S1-3**). After 2030, when other technologies, such as water electrolysis with greater cost reduction potential, become mature and take up the market share previously occupied by BECCS, the life cycle GHG intensity mix slightly increases but is still lower than that in 2025. The yearly life cycle GHG emission intensities of individual H_2 production pathways are shown in **Table S10.**

Supplementary Note 5: H_2 consumption cap

For the commercial and residential building sectors, we apply a cap of H_2 consumption that represents the theoretically highest H_2 blending volume in current natural gas pipelines. Currently, the H_2 blending ratio with the natural gas pipeline is 20% by volume in pilot locations (5). We assume that in the most promising case, when the blending ratio reaches the maximum, 20% of the natural gas consumption would be replaced by H_2. This value serves as the cap for each year between 2025 and 2050. We choose the year 2023 as the baseline year to derive the natural gas consumption in commercial and residential building sectors, as yearly natural gas consumption remains relatively stable from historical records (6). The H_2 consumption ceiling is shown in **Table S8**. The ratio represents the yearly growth of applying H_2 into natural gas pipelines. When the ratio reaches 1, 20% by the volume of natural gas consumed by residential and commercial sectors will be substituted by H_2.

Supplementary Note 6: LCA sensitivity analysis

We conducted a sensitivity analysis to understand the impacts of main parameters, including input-output coefficients, emission factors, and CCS rate, on the life cycle GHG emissions of various H_2 production pathways. Each parameter was tested within a ±20% range of its baseline value. **Fig. S10** shows the sensitivity analysis results of biomass gasification, biomass gasification CCS, coal gasification CCS, and NG SMR pathways. The sensitivity analysis of water electrolysis is not included in the analysis as electricity is its main energy source, thus the main contributor to the GHG emissions (7).

For biomass gasification, the parameter with the greatest influence on the final LCA result is the IO coefficient of biomass, followed by the IO of electricity and IO of natural gas. As biogenic carbon is considered carbon neutral in GREET, the emission factor of biomass includes biomass cultivation and transportation. For biomass gasification with CCS, the CCS rate shows the greatest impact. For coal gasification with CCS, the non-combustion emission factor of coal is the most influential parameter affecting the final LCA results. The non-combustion emission factor represents all process-related emissions, excluding those from fuel combustion. This non-combustion emission factor is available in GREET (4). The second key parameter is the CCS rate, followed by the IO factor of coal and the IO of natural gas. The coal upstream emission factor includes the coal mining and transportation to the facility.

Supplementary Note 7: H_2 leakage and losses during transport

We do not include H_2 leakage in our emissions accounting for the transmission and distribution of centrally produced H_2. This approach is consistent with several previous studies (8–10) that either exclude H_2 leakage due to the absence of standardized emission factors or find its contribution to life cycle greenhouse gas emissions to be relatively limited under typical assumptions. For example, the California fuel life cycle assessment framework explicitly assumes that H_2 leakage during production is negligible and therefore does not include it in calculations (11). One study that accounts for H_2 leakage typically applies low leakage rates, such as 0.5% per one thousand kilometers of pipeline, and reports that the resulting increase in global warming potential is minimal (12). A recent study (9) focusing on the Texas H_2 hub assumes a leakage rate of 0.3% over four hundred kilometers of pipeline transport and 1.6% for heavy-duty transportation applications. The higher leakage in transportation applications is attributed to the more frequent handling of H_2 during refueling, the use of high-pressure storage systems, and the more complex nature of vehicle-based infrastructure. This study found that while the effect of H_2 leakage on life cycle GHG is modest in most end-use sectors, such as steelmaking, it can significantly diminish the climate benefits of H_2 in heavy-duty transportation.

Given that pipeline-based delivery represents the dominant mode of H_2 distribution in our scenarios and that our study focuses on industrial end uses with more centralized infrastructure, including H_2 leakage, will not alter our main findings. We recognize that the indirect climate effects of H_2 leakage, including its role in atmospheric chemistry and radiative forcing, represent an important area for future research (13). Future researchers should explore the possibility of incorporating H_2 leakage into prospective life cycle assessments when more data and standardized modeling practices become available.

Supplementary Note 8: H_2 production cost assumptions

The GCAM model uses cost assumptions for H_2 production based on the National Renewable Energy Laboratory (NREL) H2A Production Models (14). These models provide detailed techno-economic data for various H_2 production technologies under "current" and "future" technology cases. GCAM adopts the H2A current case as the base-year input and extrapolates costs forward to 2050 using annual improvement rates that are designed to converge with H2A's future case projections. For all H_2 supply pathways, GCAM separates the total cost into two parts: (1) a non-energy component representing capital and operating costs, and (2) an energy cost (15). The H_2 production costs for all pathways included in this study are provided in **Fig. S1-3**. While this Note primarily focuses on capital and operating cost assumptions, energy costs in GCAM are calculated by multiplying the energy input requirements (e.g., EJ kg^{-1} H_2) for each production pathway by the region- and year-specific prices of electricity or fuel. These energy prices are internally projected by the model based on supply-demand dynamics. Therefore, the energy cost component varies across regions and scenarios, but its calculation is consistently applied to all H_2 production pathways.

For NG SMR, which is a mature and cost-effective technology, only small efficiency gains and cost declines are assumed in GCAM. GCAM also includes SMR with carbon capture and storage (CCS) as a low-carbon H_2 option. While SMR with CCS has higher initial costs than conventional SMR, due to additional capture equipment and reduced energy efficiency, both technologies are modeled with similar cost decline trajectories in GCAM, leading to a narrowing cost gap over time (15).

For water electrolysis, GCAM applies significant cost declines to reflect expected advances in electrolyzer design, manufacturing scale, and efficiency. Electrolyzer capital costs fall substantially through 2040 (15). For wind-power and solar—power electrolysis, the GCAM model incorporates the cost of renewable generation for energy cost, adjusted by regional capacity factors. Using NREL's Annual Technology Baseline data (16), the cost of solar PV panels or wind turbines is combined with the electrolyzer system.

For biomass gasification, GCAM assumes cost reductions based on H2A's future projections. Biomass with CCS is included as a carbon-negative pathway. Its non-energy cost related to CCS is derived by applying cost and efficiency penalties based on similar systems in the power sector (15).

Overall, based on the aforementioned GCAM's default assumptions, between 2025 and 2050, the non-energy costs of wind-powered and solar-powered electrolysis are projected to decline by 35.3% and 46.9%, respectively, while the costs of biomass gasification and BECCS decrease more modestly by 1.5% and 3.7% respectively, as shown in **Fig. S1-3**.

Supplementary Note 9: GCAM choice model

GCAM uses the choice model to simulate market competition among different energy technologies and fuels (17,18). Two main choice models are adopted in GCAM, logit model and modified logit model. These models calculate the market shares for different technologies based on relative costs and market preferences.

The logit model (19) is detailed in the following equation (*S6*):

$$share_i = \frac{\alpha_i * \exp\left(\beta p_i\right)}{\sum_{j=1}^{N} \alpha_j * \exp\left(\beta p_j\right)} \qquad (S6)$$

where *i, j* are different technology choice options. *α* represents the shareweight parameter. It is a parameter used to (1) calibrate model outputs to historical data by capturing regional preferences and structural factors, and (2) control the market entry of new technologies by assigning them low initial values that increase over time, enabling gradual adoption. β is the logit coefficient, determines the sensitivity of market share allocation to cost differences. *p* represents the sum of the energy and non-fuel costs.

The modified logit model (20) is detailed in the following equation (*S7*):

$$share_i = \frac{\alpha_i * p_i^{\gamma}}{\sum_i \alpha_j * p_j^{\gamma}} \qquad (S7)$$

where *i, j, α ,*β*, p* have the same meaning with the logic model. γ is the logit exponent that graduates the level of penetration of different fuels or technologies.

The full documentation of both choice models can be found in the GCAM online documentation (https://github.com/JGCRI/gcam-doc/blob/gh-pages/choice.md).

Supplementary Note 10: Scenario setting

Five basic scenarios with different policy incentives for clean H_2 production are analyzed, including a Baseline scenario and four scenarios with varying support levels for H_2, i.e., BAU, Present, Water+, and Biomass+. Each scenario is combined with three sets of additional considerations, including: a) the availability of Bio-H_2 (with and without Bio-H_2 from biomass gasification), b) the development of water electrolysis technology, and c) varying levels of national economy-wide carbon prices, as shown in **main text Table 1**. While the main results and conclusions of this study are based on the no carbon price scenario which reflects the current U.S. policy context, we extend the analysis to higher carbon pricing conditions to examine whether and how the findings may change under more stringent climate policy environments. In our model, the development level of water electrolysis is reflected by the costs of wind turbines, solar panels, and electrolyzers (**SI Appendix, Table S9**).

The Present scenario applies the current IRA 45V tax incentive allocation based on the life cycle GHG emissions of H_2 production. Four incentive categories of IRA 45V are shown in **SI Appendix, Table S13**. The Water+ and Biomass+ scenarios include extra incentives to promote the development of green H_2. In the Water+ scenario, the incentives for solar power and wind power electrolysis are elevated to the highest category \$3 kg^{-1} H_2 produced from centralized and decentralized production. In the Biomass+ scenario, the incentive for H_2 biomass gasification centralized production is increased from \$0.75 kg^{-1} H_2 to \$1 kg^{-1} H_2. The incentive for biomass gasification onsite production increased from \$0.6 kg^{-1} H_2 to \$1.5 kg^{-1} H_2. All values are reported in 2023 dollars. As the incentive for biomass gasification with CCS is already being credited with the highest category based on its current life cycle emissions in IRA 45V (21), we add additional incentives equal to carbon capture credits from 45Q. It is important to note that currently, H_2 plants cannot receive credits from both 45V and 45Q. We use the 45Q incentive value as a benchmark to set the increased value. For details, see **SI Appendix, Table S6**.

The carbon price serves as a proxy for a range of domestic mitigation policies that influence both supply- and demand-side dynamics within the H_2 sector. The higher carbon price reflects a more stringent policy on GHG mitigation (22,23). We apply a 2025-2050 CO_2 price trajectory that is consistent with the net-zero emission target (24). As the literature only provides carbon prices till 2030, we adopt an upper limit of CO_2 price (25) in 2050 and the prices in between years follow linear extrapolation. The carbon prices of each tonne of carbon used in this study are documented in **main text Table 2**.

SI Figures

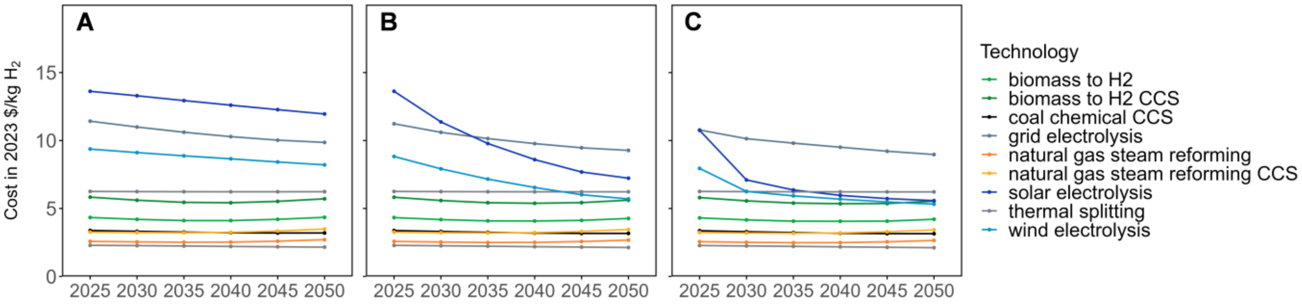

Fig. S1. H_2 central production prices under BAU scenario.

(**A**) Low water electrolysis development, (**B**) Medium water electrolysis development, and (**C**) Advanced water electrolysis development.

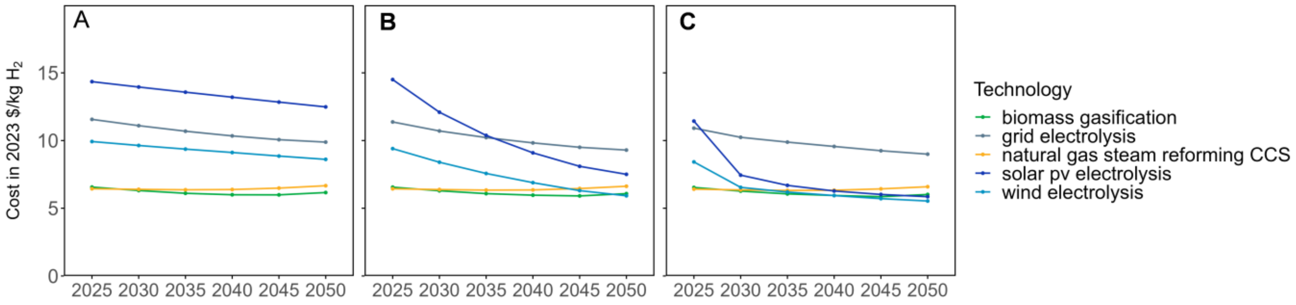

Fig. S2. H_2 onsite production prices under BAU scenario.

(**A**) Low water electrolysis development, (**B**) Medium water electrolysis development, and (**c**) Advanced water electrolysis development.

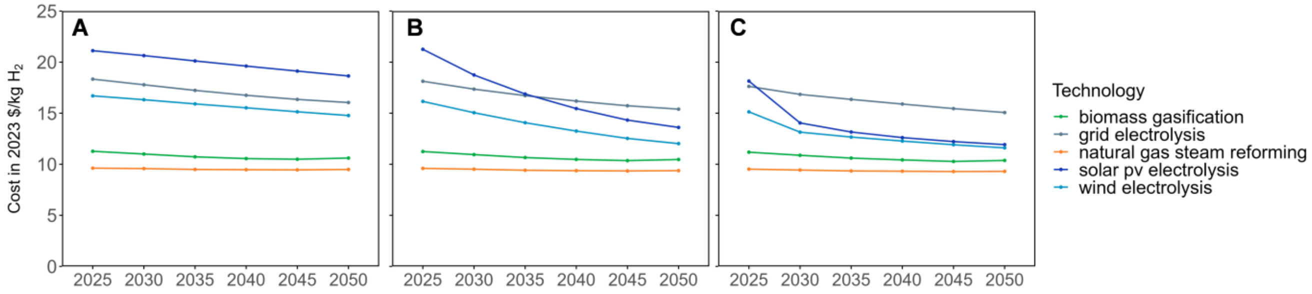

Fig. S3. H_2 onsite (forecourt) production prices under BAU scenario.

(**A**) Low water electrolysis development, (**B**) Medium water electrolysis development, and (**C**) Advanced water electrolysis development. The onsite (forecourt) production is also classified as distributed H_2 production (26). Our analysis considers both onsite and onsite (forecourt) as onsite H_2 production. Onsite and onsite (forecourt) have different end-use sectors, thus different additional transportation and distribution costs. H_2 from onsite (forecourt) production adopts H_2 dispensing while the onsite production adopts H_2 delivery. Dispensing is generally used for hydrogen that is held at high pressure and/or low temperatures for end-use purposes (e.g., vehicles and other mobile applications), whereas delivery is used for stationary sources where storage volume is not as constrained (e.g., buildings and industrial facilities) (26).

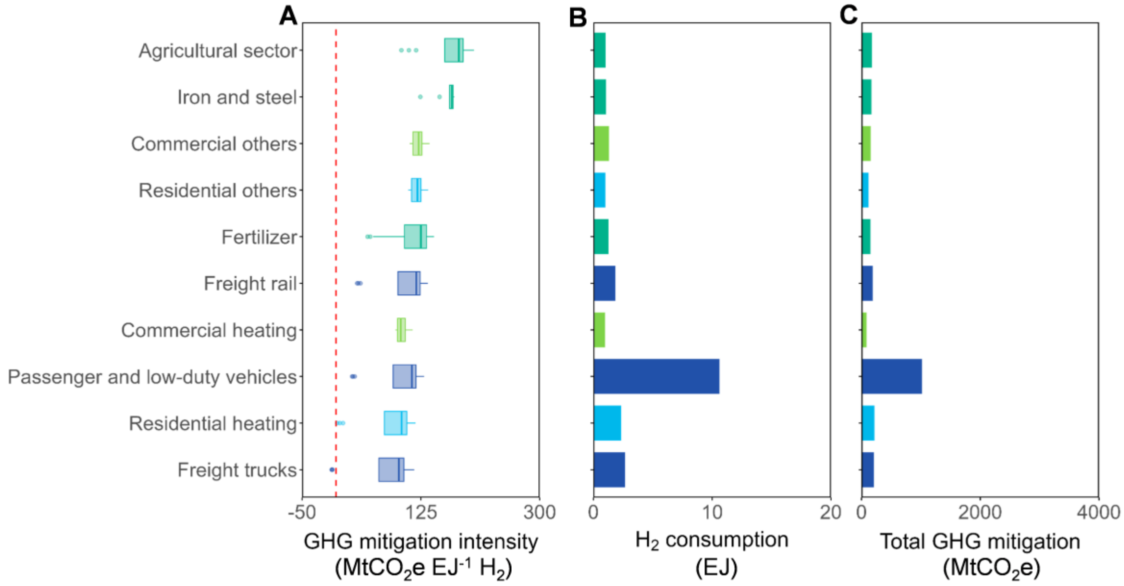

Fig. S4. Sectoral H_2 consumption and mitigation potential mismatch (low carbon price scenario).

(**A**) The greenhouse gas (GHG) mitigation potential of 1 EJ H_2 in end-use sectors. The error bars reflect the uncertainty from various scenario settings. Only sectors with hydrogen consumption larger than 3% of the total hydrogen production are selected. (**B**) The sector-wise cumulative H_2 consumption averaged from all scenarios, including the industrial sector, the commercial sector, the residential sector, and the transportation sector. Values are cumulative H_2 consumption from 2025 to 2050. (**C**) The sector-wise cumulative GHG mitigation averaged from all scenarios. Values are cumulative GHG mitigated from 2025 to 2050. Note that the Baseline scenarios here are scenarios where there is no H_2 production but with the same carbon price. This setting enables the quantification of mitigation caused only by the introduction of H_2, thus eliminating the mitigation caused by the other decarbonization efforts.

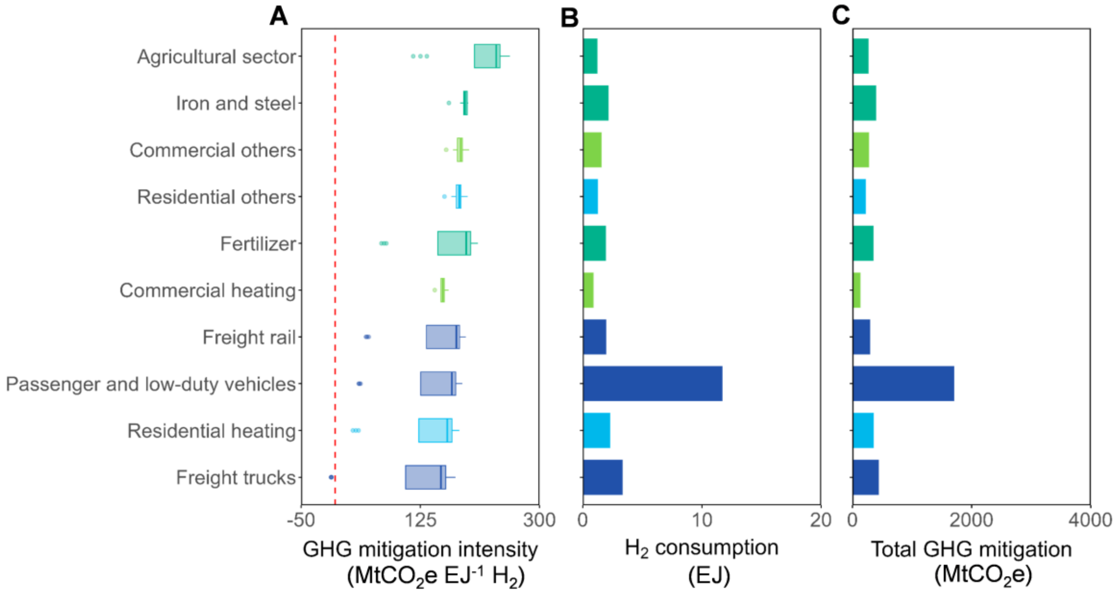

Fig. S5. Sectoral H_2 consumption and mitigation potential mismatch (medium carbon price scenario).

(**A**) The greenhouse gas (GHG) mitigation potential of 1 EJ H_2 in end-use sectors. The error bars reflect the uncertainty from various scenario settings. Only sectors with hydrogen consumption larger than 3% of the total hydrogen production are selected. (**B**) The sector-wise cumulative H_2 consumption averaged from all scenarios, including the industrial sector, the commercial sector, the residential sector, and the transportation sector. Values are cumulative H_2 consumption from 2025 to 2050. (**C**) The sector-wise cumulative GHG mitigation averaged from all scenarios. Values are cumulative GHG mitigated from 2025 to 2050. Note that the Baseline scenarios here are scenarios where there is no H_2 production but with the same carbon price. This setting enables the quantification of mitigation caused only by the introduction of H_2, thus eliminating the mitigation caused by the other decarbonization efforts.

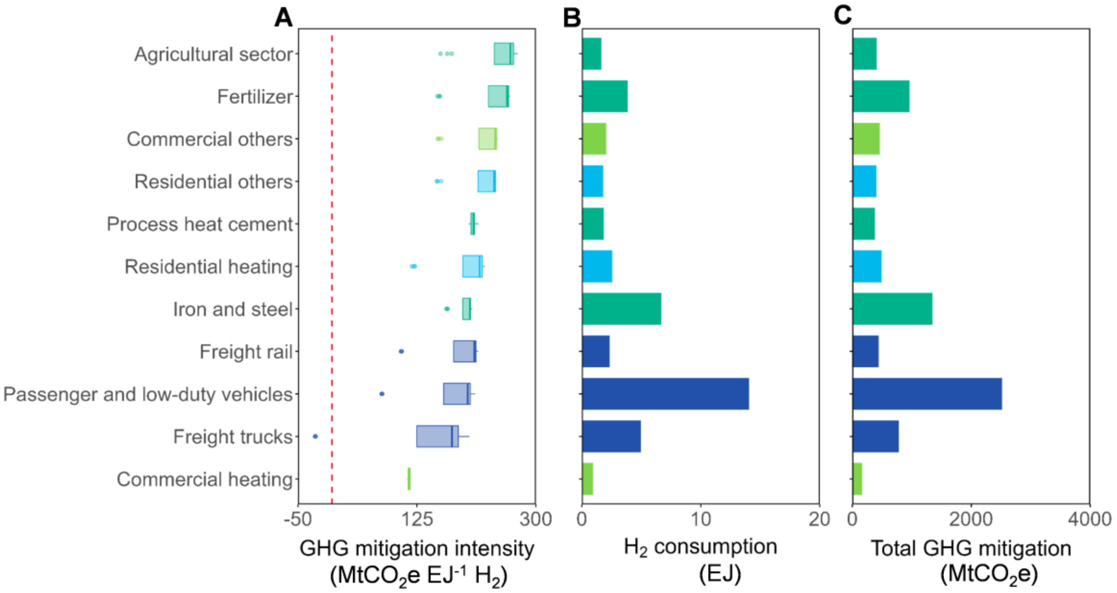

Fig. S6. Sectoral H_2 consumption and mitigation potential mismatch (high carbon price scenario).

(**A**) The greenhouse gas (GHG) mitigation potential of 1 EJ H_2 in end-use sectors. The error bars reflect the uncertainty from various scenario settings. Only sectors with hydrogen consumption larger than 3% of the total hydrogen production are selected. (**B**) The sector-wise cumulative H_2 consumption averaged from all scenarios, including the industrial sector, the commercial sector, the residential sector, and the transportation sector. Values are cumulative H_2 consumption from 2025 to 2050. (**C**) The sector-wise cumulative GHG mitigation averaged from all scenarios. Values are cumulative GHG mitigated from 2025 to 2050. Note that the Baseline scenarios here are scenarios where there is no H_2 production but with the same carbon price. This setting enables the quantification of mitigation caused only by the introduction of H_2, thus eliminating the mitigation caused by the other decarbonization efforts.

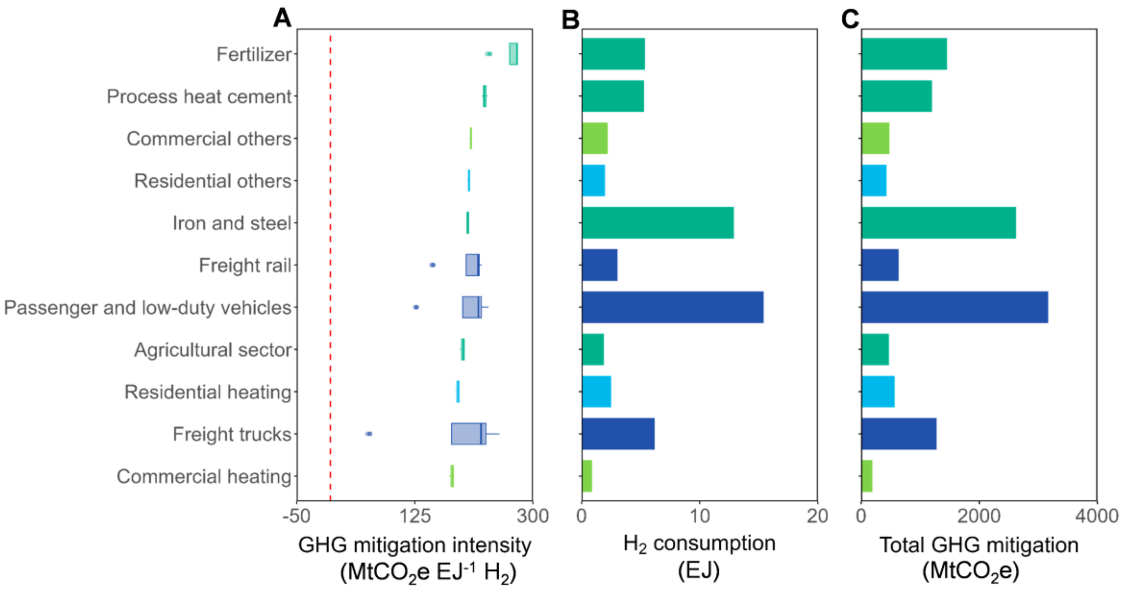

Fig. S7. Sectoral H_2 consumption and mitigation potential mismatch (extremely high carbon price scenario).

(**A**) The greenhouse gas (GHG) mitigation potential of 1 EJ H_2 in end-use sectors. The error bars reflect the uncertainty from various scenario settings. Only sectors with hydrogen consumption larger than 3% of the total hydrogen production are selected. (**B**) The sector-wise cumulative H_2 consumption averaged from all scenarios, including the industrial sector, the commercial sector, the residential sector, and the transportation sector. Values are cumulative H_2 consumption from 2025 to 2050. (**C**) The sector-wise cumulative GHG mitigation averaged from all scenarios. Values are cumulative GHG mitigated from 2025 to 2050. Note that the Baseline scenarios here are scenarios where there is no H_2 production but with the same carbon price. This setting enables the quantification of mitigation caused only by the introduction of H_2, thus eliminating the mitigation caused by the other decarbonization efforts.

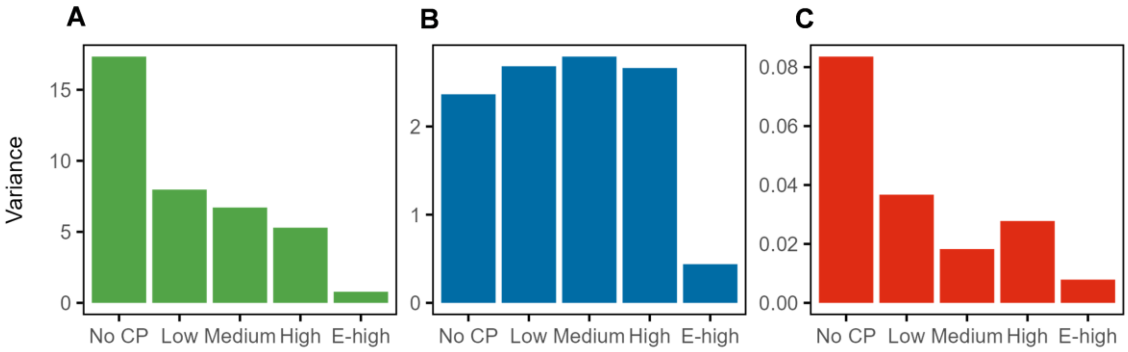

Fig. S8. Variances across different carbon price scenarios.

(**A**) Variances of average H_2 consumption. (**B**) Variances of normalized cumulative GHG mitigation potential among all sectors under varying carbon price scenarios. The sector with the highest GHG mitigation intensity is chosen as the baseline. The GHG mitigation for each sector is then normalized by dividing its GHG mitigation by that of the baseline sector. (**C**) Variances of normalized overall GHG mitigation. No CP refers to no carbon price scenario. Low, Medium, High, E-high are low, medium, high, extremely-high carbon price scenarios respectively.

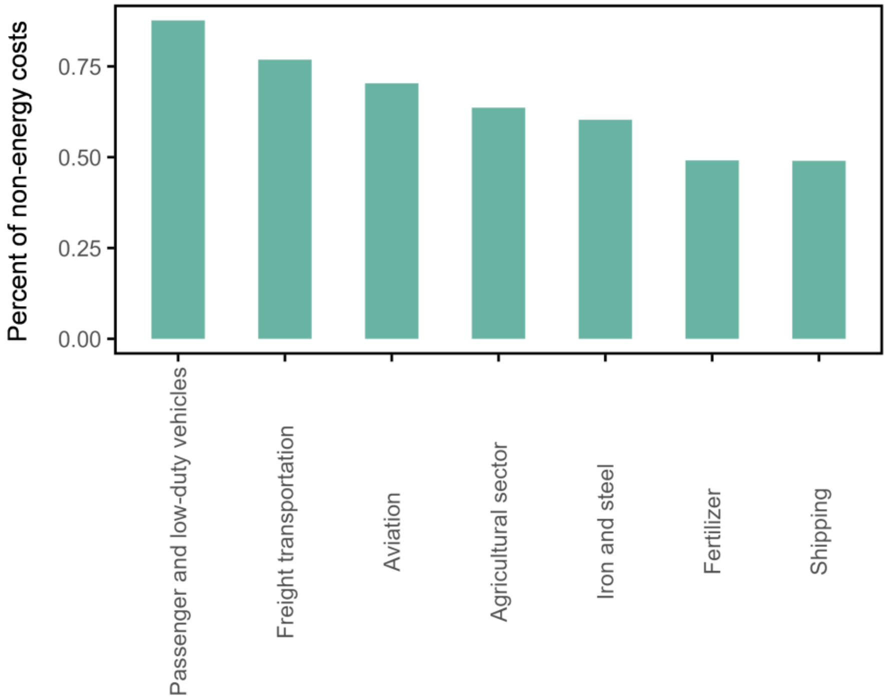

Fig. S9. Non-energy costs percent across transportation and industrial sectors.

The representative scenario here is the Present scenario with Bio-H_2 under the medium water electrolysis development.

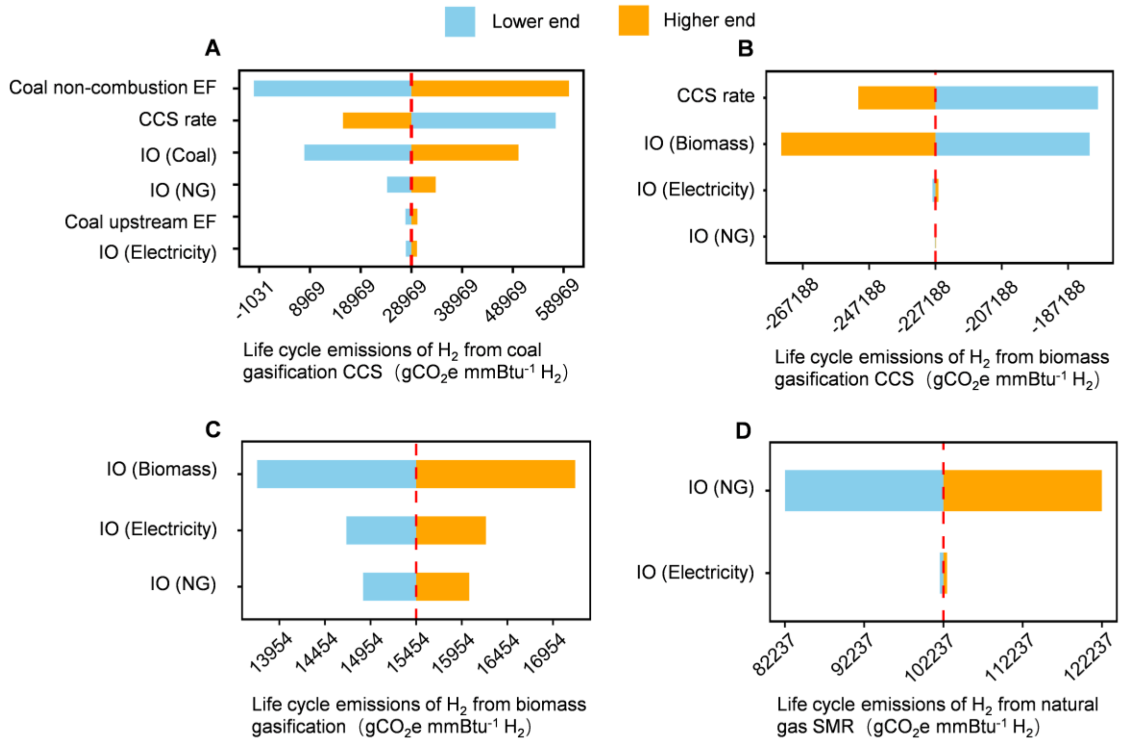

Fig. S10. Sensitivity analysis of the life cycle emission for each H_2 production pathway.

Each parameter was tested within a ±20% range of its baseline value. (**A**) Coal gasification with carbon capture and storage (CCS), (**B**) Biomass gasification with CCS, (**C**) Biomass gasification, (**D**) Natural gas steam reforming. The lower and higher ends represent a ±20% range of parameters.

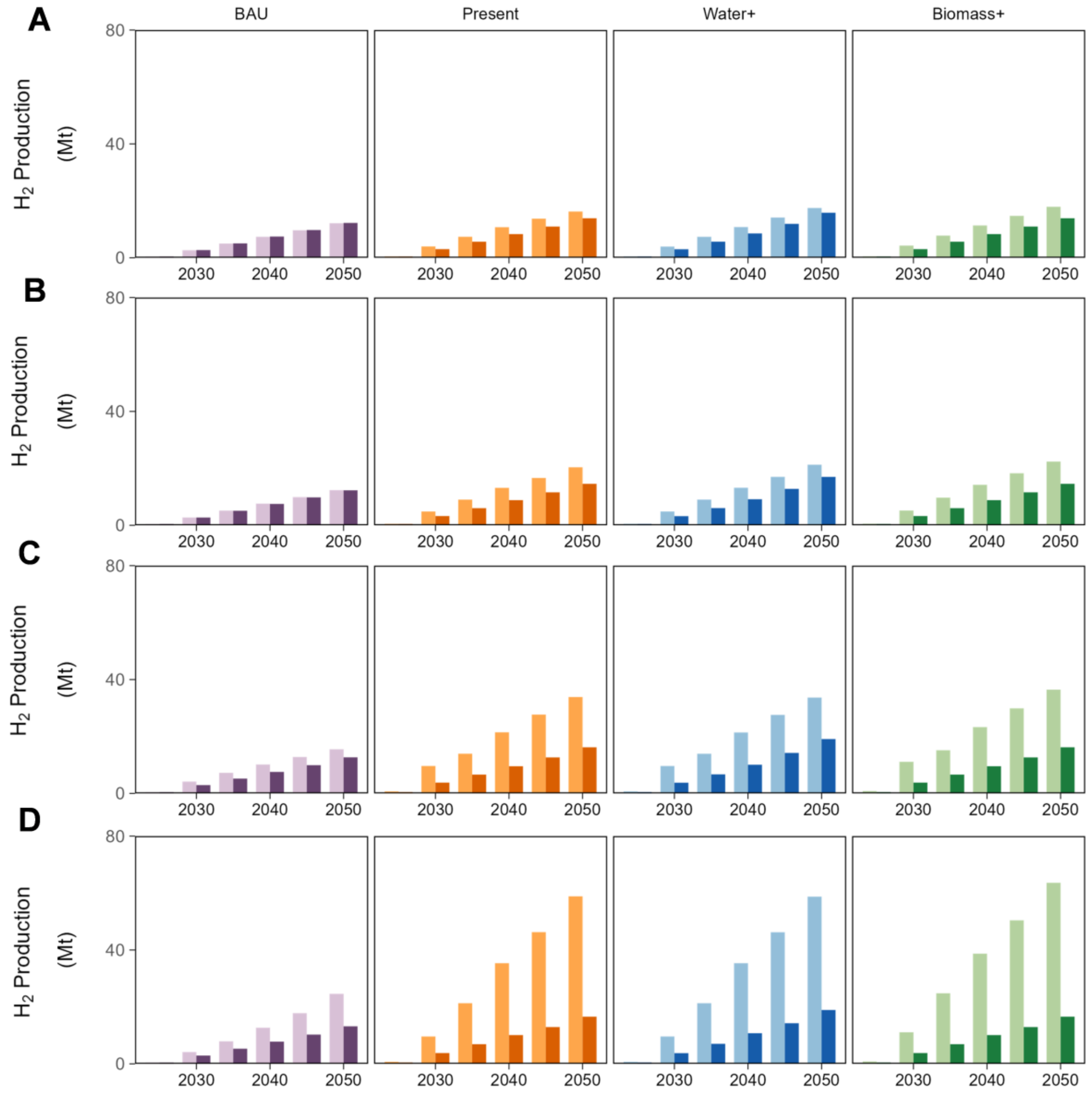

Fig. S11. H_2 production under different carbon price trajectories.

(**A**) Low carbon price scenario, (**B**) Medium carbon price scenario, (**C**) High carbon price scenario, (**D**) Extreme high carbon price scenario.

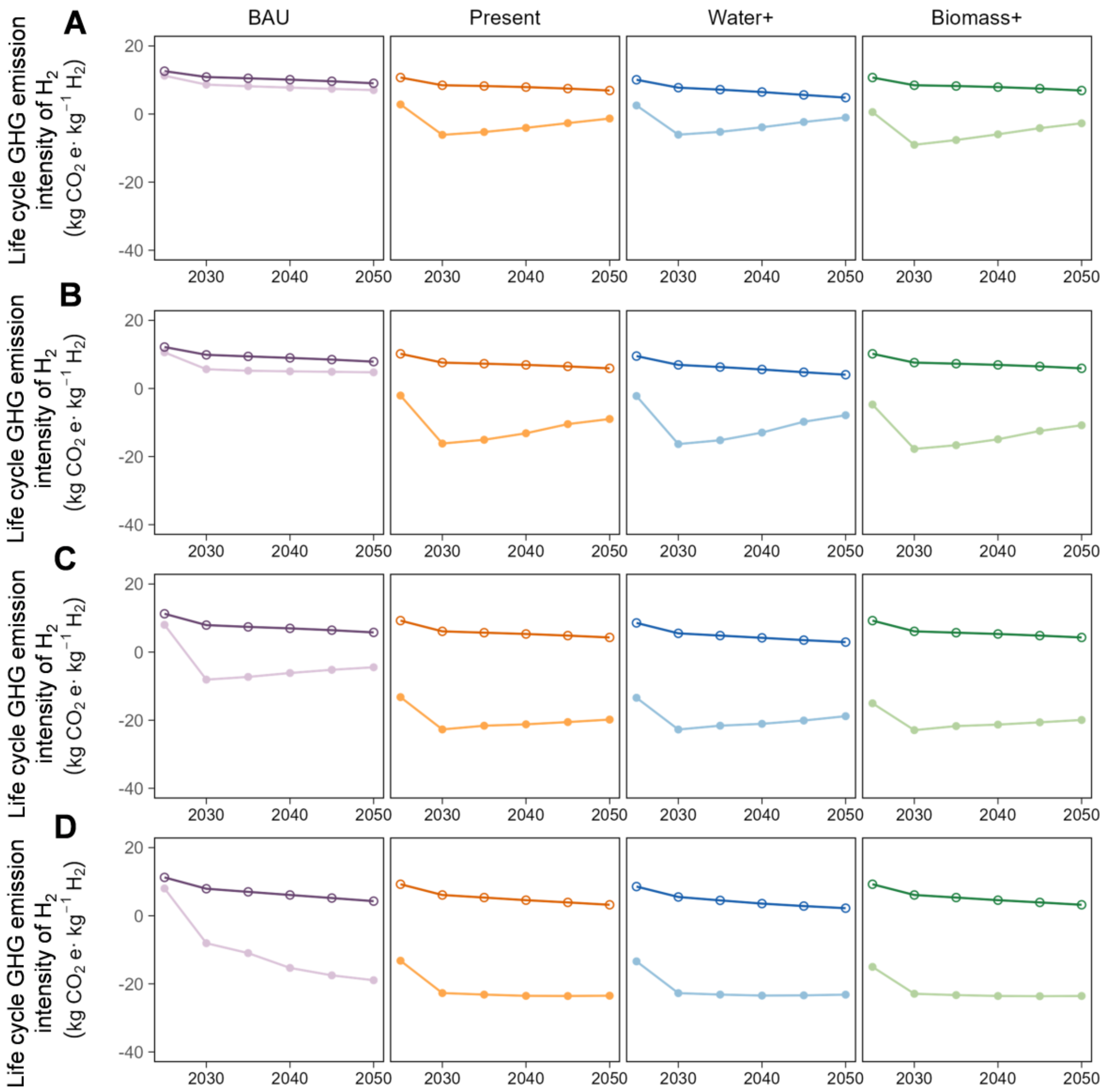

Fig. S12. H_2 production mix intensity under different carbon price trajectories.

(**A**) Low carbon price scenario, (**B**) Medium carbon price scenario, (**C**) High carbon price scenario, (**D**) Extreme high carbon price scenario.

SI Tables

Table S1. Results of cumulative GHG emission mitigation compared with no H_2 and no carbon price scenarios with medium water electrolysis development

Scenario	No carbon price	Low	Medium	High	Extremely High	Unit
Biomass+ (with Bio-H_2)	1706	11621	21822	47581	70697	Mt CO_2e
Present (with Bio-H_2)	1461	10996	20884	46252	68235	Mt CO_2e
BAU (with Bio-H_2)	607	8426	15870	36898	53811	Mt CO_2e
Water+ (with Bio-H_2)	1544	10958	20758	46033	68120	Mt CO_2e
Biomass+ (w/o Bio-H_2)	683	8487	15711	34724	48816	Mt CO_2e
Present (w/o Bio-H_2)	683	8487	15711	34724	48816	Mt CO_2e
BAU (w/o Bio-H_2)	349	8106	15315	34401	48519	Mt CO_2e
Water+ (w/o Bio-H_2)	963	8740	15947	34873	48909	Mt CO_2e

Table S2. Results of costs of various steelmaking technologies under the Present scenario with medium water electrolysis technology development in GCAM

Technology	2025	2030	2035	2040	2045	2050	Unit
BF-BOF	0.134	0.134	0.134	0.134	0.135	0.135	1975$ kg^{-1}
BF-BOF CCS	0.143	0.143	0.144	0.144	0.145	0.147	1975$ kg^{-1}
BF-BOF CCS with hydrogen	0.176	0.168	0.167	0.168	0.168	0.169	1975$ kg^{-1}
BF-BOF with hydrogen	0.166	0.158	0.158	0.158	0.158	0.159	1975$ kg^{-1}
Biomass-based	0.153	0.152	0.151	0.152	0.153	0.156	1975$ kg^{-1}
EAF with DRI	0.339	0.336	0.333	0.335	0.344	0.357	1975$ kg^{-1}
EAF with DRI CCS	0.347	0.344	0.342	0.346	0.357	0.374	1975$ kg^{-1}
Hydrogen-based DRI	0.275	0.239	0.236	0.234	0.234	0.235	1975$ kg^{-1}
EAF with scrap	0.165	0.164	0.163	0.163	0.163	0.164	1975$ kg^{-1}

*BF-BOF: blast furnace basic oxygen furnace. EAF-DRI: electric arc furnace direct reduction iron.

Table S3. Results of electricity technology baseline emission factors in 2020

Electricity generation technology	Input	IO coefficient	Total emissions (g CO_2e $mmBtu^{-1}$ electricity)
Concentrated Solar Power with Storage	Global solar resource	0.975	11052
Generation III nuclear	Generation III fuel	0.333	648
Generation II nuclear	Generation II fuel	0.333	843
Biomass (IGCC CCS)	Regional biomass	0.286	8990
Biomass (IGCC)	Regional biomass	0.333	13762
Biomass (conventional CCS)	Regional biomass	0.214	8152
Biomass (conventional)	Regional biomass	0.275	13819
Coal (IGCC CCS)	Regional coal	0.361	53589
Coal (IGCC)	Regional coal	0.420	267102
Coal (conventional pulverized CCS)	Regional coal	0.319	59738
Coal (conventional pulverized)	Regional coal	0.406	278297
Gas (CC CCS)	Wholesale gas	0.491	30625
Gas (CC)	Wholesale gas	0.571	130238
Gas (steam/CT)	Wholesale gas	0.381	195190
Geothermal	Geothermal	0.098	29442
Refined liquids (CC CCS)	Refined liquids industrial	0.454	39188
Refined liquids (CC)	Refined liquids industrial	0.561	187201
Refined liquids (steam)	Refined liquids industrial	0.368	283989
Electricity	Global solar resource	0.960	11052
Electricity	Onshore wind resource	0.960	3012

*IGCC stands for Integrated gasification combined cycle.

Table S4. Feedstock input-output coefficients of H_2 production in GCAM assumption

H_2 production pathway	Feedstock	2025	2030	2035	2040	2045	2050
NG SMR (central)	Natural gas	1.370	1.370	1.370	1.369	1.369	1.368
NG SMR (central)	Electricity	0.017	0.018	0.018	0.018	0.018	0.018
NG SMR (onsite)	Natural gas	1.332	1.314	1.297	1.280	1.263	1.246
NG SMR (onsite)	Electricity	0.193	0.200	0.208	0.217	0.217	0.217
Coal gasification (central)	Coal	1.732	1.690	1.652	1.618	1.588	1.560
Coal gasification (central)	Electricity	0.042	0.041	0.040	0.039	0.038	0.038
Water electrolysis (central)	Electricity	1.576	1.551	1.527	1.503	1.480	1.457
Water electrolysis (onsite)	Electricity	1.582	1.557	1.531	1.506	1.482	1.458
Thermal splitting (central)	Electricity	1.198	1.198	1.198	1.198	1.198	1.198
Biomass gasification (onsite & central)	Natural gas	0.039	0.034	0.030	0.026	0.025	0.025
Biomass gasification (onsite & central)	Electricity	0.029	0.029	0.028	0.028	0.028	0.028
Biomass gasification (onsite & central)	Biomass	2.164	2.149	2.134	2.119	2.104	2.090
Biomass gasification CCS (central)	Electricity	0.034	0.033	0.032	0.032	0.031	0.031
Biomass gasification CCS (central)	Biomass	2.541	2.482	2.429	2.381	2.337	2.297
Biomass gasification CCS (central)	Natural gas	0.044	0.038	0.032	0.027	0.026	0.026
Coal gasification CCS (central)	Electricity	0.042	0.041	0.040	0.039	0.038	0.038
Coal gasification CCS (central)	Coal	1.732	1.690	1.652	1.618	1.588	1.560

Table S5. Results of primary fuels emission factors in Present 45V scenario with medium water electrolysis development and with Bio-H_2

Fuel EF ($t\ CO_2e\ PJ^{-1}$)	Year	No carbon price	Low	Medium	High	Extremely high
Biomass	2025	2936.1	2936.1	2936.1	2936.1	2936.1
Biomass	2030	2843.4	2843.4	2843.4	2843.4	2843.4
Biomass	2035	2793.2	2793.2	2793.2	2793.2	2793.2
Biomass	2040	2791.2	2791.2	2791.2	2791.2	2791.2
Biomass	2045	2789.1	2789.1	2789.1	2789.1	2789.1
Biomass	2050	2725.9	2725.9	2725.9	2725.9	2725.9
Gas	2025	69114.5	68768.5	68454.1	67856.4	67857.3
Gas	2030	69007.5	68354.8	68038.7	67533.4	67534.2
Gas	2035	68990.8	68324.5	67703.3	66666.6	66778.3
Gas	2040	68980.4	68262.7	67610.4	66049.3	64933.2
Gas	2045	68860.4	68027.2	67320.4	65898.1	64337.5
Gas	2050	68780.0	67719.5	66899.1	65441.8	63387.7
Coal	2025	100805.8	100805.8	100805.8	100805.8	100805.8
Coal	2030	100733.3	100733.3	100733.3	100733.3	100733.3
Coal	2035	100718.6	100718.6	100718.6	100718.6	100718.6
Coal	2040	100714.4	100714.4	100714.4	100714.4	100714.4
Coal	2045	100709.9	100709.9	100709.9	100709.9	100709.9
Coal	2050	100701.4	100701.4	100701.4	100701.4	100701.4
Refined liquids	2025	78208.5	78184.1	78157.3	78079.0	78079.2
Refined liquids	2030	77935.6	77859.8	77761.0	71047.4	71076.4
Refined liquids	2035	78103.1	78009.9	77874.9	70073.2	58810.4
Refined liquids	2040	76698.5	76078.6	75154.3	63958.0	43738.8
Refined liquids	2045	71952.2	69274.1	66142.3	55263.1	32395.4
Refined liquids	2050	68204.1	63882.2	58924.6	46215.8	23996.7
Electricity	2025	125412.2	123298.7	121034.0	114313.7	114327.3
Electricity	2030	119550.9	113009.9	105365.8	74919.7	74971.2
Electricity	2035	114524.0	104865.4	94875.7	65303.2	54146.5
Electricity	2040	108915.3	96644.6	85144.2	56345.7	36501.4
Electricity	2045	105819.5	90801.1	77565.2	49036.0	25790.3
Electricity	2050	103500.0	86227.8	71440.6	43252.7	19119.0

Table S6. H_2 Tax incentives set up (unit: \$1975 GJ^{-1} H_2)

Production pathways	Present	Water+	Biomass+
Biomass (central)	-1.104	-1.104	-1.472
Biomass CCS (central)	-4.417	-4.417	Around -4.8*
Nuclear	-4.417	-4.417	-4.417
Solar (onsite & central)	-0.883	-4.417	-0.883
Wind (onsite & central)	-1.472	-4.417	-1.472
Coal CCS	-0.883	-0.883	-0.883
NG	0	0	0
NG CCS (central)	-0.883	-0.883	-0.883
Grid	0	0	0
Biomass (onsite)	-0.883	-0.883	-2.208

*The value varies by year according to the amount of captured carbon.

The tax incentives are determined based on IRA 45V (21), which defines five incentive categories (as outlined in the **main text Table 2**). In the Water+ and Biomass+ scenarios, we simulate the promotion of water electrolysis and biomass gasification by increasing their tax incentives to the next higher category compared to the Present scenario. For biomass gasification with CCS, which already qualifies for the highest incentive category, we use the 45Q incentive (27) as a benchmark value to represent additional policy support for BECCS development. We apply a \$12 base incentive, which is the lowest base credit value, for every tonne of CO_2 captured by BECCS. To ensure consistency, the magnitude of incentive increases for onsite water electrolysis in Water+ is matched by increasing the tax incentives in Biomass+ to half of the highest incentive category.

Table S7. Breakdown of 2022 US refiner and blender net production of traditional oil refined products

Products	Volume[5]%	Weighted energy (Btu gal^{-1})	Energy%	Only liquid products
Gasoline	0.53	59463	53.6%	56%
Diesel	0.26	33397	30.1%	32%
Conventional jet fuel	0.08	9945	9.0%	9%
Pet coke	0.04	574	0.5%	Not considered
Still gas	0.03	260	0.2%	Not considered
Natural gas liquids	0.03	2511	2.3%	2%
Asphalt	0.03	4740	4.3%	Not considered

Table S8. H_2 consumption cap

	2025	2030	2035	2040	2045	2050	
Ratio	0.15	0.30	0.45	0.60	0.75	1.00	
Residential	0.319	0.638	0.958	1.277	1.596	2.128	Mt
Commercial	0.203	0.406	0.610	0.813	1.016	1.355	Mt

Table S9. Water electrolysis cost assumptions in GCAM (unit: \$1975 GJ^{-1} electricity)

Year	Water electrolysis development	Solar PV	Wind turbine	Electrolyzer (solar-power)	Electrolyzer (wind-power)
2025	Advanced	7.45	6.19	7.69	4.18
2030	Advanced	5.46	5.52	4.33	2.35
2035	Advanced	4.38	5.04	4.33	2.35
2040	Advanced	3.81	4.68	4.33	2.35
2045	Advanced	3.47	4.39	4.33	2.35
2050	Advanced	3.26	4.18	4.33	2.35
2025	Low	9.87	7.43	9.32	5.08
2030	Low	9.66	7.21	9.02	4.92
2035	Low	9.47	7.03	8.73	4.76
2040	Low	9.29	6.87	8.44	4.60
2045	Low	9.12	6.73	8.15	4.44
2050	Low	8.96	6.60	7.85	4.28
2025	Medium	10.10	6.63	9.32	5.08
2030	Medium	8.24	6.07	7.85	4.28
2035	Medium	7.14	5.62	6.62	3.62
2040	Medium	6.45	5.27	5.58	3.05
2045	Medium	5.99	4.99	4.70	2.57
2050	Medium	5.69	4.77	4.33	2.35

Cost assumptions for advanced, medium, and low solar PV and wind turbines are directly derived from the default GCAM model. The cost assumptions for electrolyzers in the medium water electrolysis development scenario are available in GCAM. For advanced water electrolysis development, we assume an aggressive cost reduction, with electrolyzer costs reaching the 2050 level by 2030. In the low water electrolysis development scenario, electrolyzer costs are assumed to reach the 2050 level by 2100.

Table S10. Life cycle GHG emission intensity of individual H_2 production pathways in each year (unit: Mt CO_2e EJ^{-1} H_2)

Location	Technology	Year	Present	Water+	Biomass+	BAU
Central production	Biomass gasification CCS	2025	-219.389	-219.389	-219.389	-219.389
Central production	Biomass gasification CCS	2030	-215.331	-215.331	-215.331	-215.331
Central production	Biomass gasification CCS	2035	-211.734	-211.733	-211.734	-211.734
Central production	Biomass gasification CCS	2040	-208.403	-208.403	-208.403	-208.404
Central production	Biomass gasification CCS	2045	-205.325	-205.324	-205.324	-205.326
Central production	Biomass gasification CCS	2050	-202.435	-202.433	-202.434	-202.437
Central production	Biomass gasification	2025	15.538	15.538	15.538	15.538
Central production	Biomass gasification	2030	14.65	14.65	14.65	14.649
Central production	Biomass gasification	2035	13.829	13.829	13.829	13.829
Central production	Biomass gasification	2040	13.182	13.183	13.182	13.182
Central production	Biomass gasification	2045	12.67	12.671	12.67	12.669
Central production	Biomass gasification	2050	12.415	12.417	12.415	12.413
Central production	Coal chemical CCS	2025	29.11	29.11	29.11	29.11
Central production	Coal chemical CCS	2030	27.46	27.46	27.46	27.46
Central production	Coal chemical CCS	2035	25.929	25.929	25.929	25.928
Central production	Coal chemical CCS	2040	24.643	24.643	24.643	24.642
Central production	Coal chemical CCS	2045	23.441	23.442	23.441	23.44
Central production	Coal chemical CCS	2050	22.41	22.413	22.411	22.408
Central production	Grid electrolysis	2025	212.851	212.851	212.851	212.851
Central production	Grid electrolysis	2030	197.917	197.92	197.919	197.916
Central production	Grid electrolysis	2035	185.723	185.734	185.728	185.715
Central production	Grid electrolysis	2040	175.152	175.176	175.162	175.127
Central production	Grid electrolysis	2045	163.993	164.041	164.011	163.944
Central production	Grid electrolysis	2050	156.858	156.957	156.885	156.775
Central production	Natural gas steam reforming CCS	2025	28.033	28.033	28.034	28.031
Central production	Natural gas steam reforming CCS	2030	27.636	27.636	27.637	27.634
Central production	Natural gas steam reforming CCS	2035	27.122	27.122	27.127	27.112
Central production	Natural gas steam reforming CCS	2040	26.747	26.749	26.755	26.733
Central production	Natural gas steam reforming CCS	2045	26.371	26.374	26.381	26.353
Central production	Natural gas steam reforming CCS	2050	25.893	25.897	25.91	25.87
Central production	Natural gas steam reforming	2025	97.003	97.003	97.004	97.001
Central production	Natural gas steam reforming	2030	96.899	96.899	96.9	96.897
Central production	Natural gas steam reforming	2035	96.642	96.642	96.647	96.632
Central production	Natural gas steam reforming	2040	96.522	96.523	96.53	96.508
Central production	Natural gas steam reforming	2045	96.398	96.402	96.409	96.381
Central production	Natural gas steam reforming	2050	96.149	96.153	96.165	96.126
Central production	Nuclear thermal splitting	2025	1.11	1.11	1.11	1.11
Central production	Nuclear thermal splitting	2030	1.06	1.06	1.06	1.06

Central production	Nuclear thermal splitting	2035	0.955	0.955	0.955	0.955
Central production	Nuclear thermal splitting	2040	0.931	0.931	0.931	0.931
Central production	Nuclear thermal splitting	2045	0.921	0.921	0.921	0.921
Central production	Nuclear thermal splitting	2050	0.911	0.911	0.911	0.911
Central production	Solar-power electrolysis	2025	22.901	22.901	22.901	22.901
Central production	Solar-power electrolysis	2030	16.854	16.868	16.813	16.969
Central production	Solar-power electrolysis	2035	15.584	15.6	15.535	15.706
Central production	Solar-power electrolysis	2040	15.104	15.122	15.053	15.232
Central production	Solar-power electrolysis	2045	14.772	14.78	14.724	14.904
Central production	Solar-power electrolysis	2050	14.485	14.47	14.446	14.608
Central production	Wind-power electrolysis	2025	10.688	10.688	10.688	10.688
Central production	Wind-power electrolysis	2030	6.143	6.156	6.101	6.258
Central production	Wind-power electrolysis	2035	5.828	5.845	5.779	5.951
Central production	Wind-power electrolysis	2040	5.718	5.736	5.667	5.847
Central production	Wind-power electrolysis	2045	5.642	5.649	5.593	5.773
Central production	Wind-power electrolysis	2050	5.609	5.594	5.571	5.732
Forecourt production	Grid electrolysis	2025	213.876	213.876	213.876	213.876
Forecourt production	Grid electrolysis	2030	198.73	198.732	198.731	198.728
Forecourt production	Grid electrolysis	2035	186.354	186.365	186.359	186.346
Forecourt production	Grid electrolysis	2040	175.623	175.648	175.634	175.598
Forecourt production	Grid electrolysis	2045	164.319	164.367	164.336	164.27
Forecourt production	Grid electrolysis	2050	157.059	157.158	157.085	156.976
Forecourt production	Grid electrolysis	2025	213.876	213.876	213.876	213.876
Forecourt production	Grid electrolysis	2030	198.73	198.732	198.731	198.728
Forecourt production	Grid electrolysis	2035	186.354	186.365	186.359	186.346
Forecourt production	Grid electrolysis	2040	175.623	175.648	175.634	175.598
Forecourt production	Grid electrolysis	2045	164.319	164.367	164.336	164.27
Forecourt production	Grid electrolysis	2050	157.059	157.158	157.085	156.976
Forecourt production	Natural gas steam reforming	2025	116.909	116.909	116.91	116.908
Forecourt production	Natural gas steam reforming	2030	116.266	116.267	116.267	116.265
Forecourt production	Natural gas steam reforming	2035	114.591	114.592	114.596	114.581
Forecourt production	Natural gas steam reforming	2040	113.264	113.269	113.273	113.248
Forecourt production	Natural gas steam reforming	2045	111.889	111.898	111.901	111.866
Forecourt production	Natural gas steam reforming	2050	109.902	109.919	109.921	109.87
Onsite production	Grid electrolysis	2025	213.876	213.876	213.876	213.876
Onsite production	Grid electrolysis	2030	198.73	198.732	198.731	198.728
Onsite production	Grid electrolysis	2035	186.354	186.365	186.359	186.346
Onsite production	Grid electrolysis	2040	175.623	175.648	175.634	175.598
Onsite production	Grid electrolysis	2045	164.319	164.367	164.336	164.27
Onsite production	Grid electrolysis	2050	157.059	157.158	157.085	156.976
Onsite production	Natural gas steam reforming CCS	2025	28.033	28.033	28.034	28.031

Onsite production	Natural gas steam reforming CCS	2030	27.636	27.636	27.637	27.634
Onsite production	Natural gas steam reforming CCS	2035	27.122	27.122	27.127	27.112
Onsite production	Natural gas steam reforming CCS	2040	26.747	26.749	26.755	26.733
Onsite production	Natural gas steam reforming CCS	2045	26.371	26.374	26.381	26.353
Onsite production	Natural gas steam reforming CCS	2050	25.893	25.897	25.91	25.87
Onsite production	Solar-power electrolysis	2025	17.147	17.147	17.147	17.147
Onsite production	Solar-power electrolysis	2030	15.393	15.393	15.393	15.393
Onsite production	Solar-power electrolysis	2035	14.1	14.1	14.1	14.1
Onsite production	Solar-power electrolysis	2040	13.605	13.605	13.605	13.605
Onsite production	Solar-power electrolysis	2045	13.261	13.261	13.261	13.261
Onsite production	Solar-power electrolysis	2050	12.918	12.918	12.918	12.918
Onsite production	Wind-power electrolysis	2025	4.875	4.875	4.875	4.875
Onsite production	Wind-power electrolysis	2030	4.637	4.637	4.637	4.637
Onsite production	Wind-power electrolysis	2035	4.311	4.311	4.311	4.311
Onsite production	Wind-power electrolysis	2040	4.194	4.194	4.194	4.194
Onsite production	Wind-power electrolysis	2045	4.113	4.113	4.113	4.113
Onsite production	Wind-power electrolysis	2050	4.031	4.031	4.031	4.031
Forecourt production	Solar-power electrolysis	2025	17.147	17.147	17.147	17.147
Forecourt production	Solar-power electrolysis	2030	15.393	15.393	15.393	15.393
Forecourt production	Solar-power electrolysis	2035	14.1	14.1	14.1	14.1
Forecourt production	Solar-power electrolysis	2040	13.605	13.605	13.605	13.605
Forecourt production	Solar-power electrolysis	2045	13.261	13.261	13.261	13.261
Forecourt production	Solar-power electrolysis	2050	12.918	12.918	12.918	12.918
Forecourt production	Wind-power electrolysis	2025	4.875	4.875	4.875	4.875
Forecourt production	Wind-power electrolysis	2030	4.637	4.637	4.637	4.637
Forecourt production	Wind-power electrolysis	2035	4.311	4.311	4.311	4.311
Forecourt production	Wind-power electrolysis	2040	4.194	4.194	4.194	4.194
Forecourt production	Wind-power electrolysis	2045	4.113	4.113	4.113	4.113
Forecourt production	Wind-power electrolysis	2050	4.031	4.031	4.031	4.031
Onsite production	Biomass gasification	2025	24.113	24.113	24.113	24.113
Onsite production	Biomass gasification	2030	22.926	22.926	22.926	22.926
Onsite production	Biomass gasification	2035	21.914	21.914	21.914	21.914
Onsite production	Biomass gasification	2040	21.206	21.207	21.206	21.206
Onsite production	Biomass gasification	2045	20.634	20.634	20.634	20.633
Onsite production	Biomass gasification	2050	20.155	20.157	20.156	20.154
Forecourt production	Biomass gasification	2025	24.113	24.113	24.113	24.113
Forecourt production	Biomass gasification	2030	22.926	22.926	22.926	22.926
Forecourt production	Biomass gasification	2035	21.914	21.914	21.914	21.914
Forecourt production	Biomass gasification	2040	21.206	21.207	21.206	21.206
Forecourt production	Biomass gasification	2045	20.634	20.634	20.634	20.633
Forecourt production	Biomass gasification	2050	20.155	20.157	20.156	20.154

Table S11. Electricity scale factor and GHG emission intensity

Year	Output	Scale factor	GHG intensity ($g\ CO_2e\ mmBtu^{-1}$ electricity)
2020	Biomass (IGCC CCS)	1.00	8990.43
2020	Biomass (IGCC)	1.00	13761.55
2020	Biomass (conventional CCS)	1.00	8152.05
2020	Biomass (conventional)	1.00	13818.94
2020	Coal (IGCC CCS)	1.00	53589.16
2020	Coal (IGCC)	1.00	267102.22
2020	Coal (conventional pulverized CCS)	1.00	59737.68
2020	Coal (conventional pulverized)	1.00	278297.28
2020	Gas (CC CCS)	1.00	30624.50
2020	Gas (CC)	1.00	130238.07
2020	Gas (steam/CT)	1.00	195189.72
2020	Geothermal	1.00	29434.23
2020	Hydropower	1.00	2190.44
2020	Generation III nuclear	1.00	636.10
2020	Generation II nuclear	1.00	828.01
2020	Refined liquids (CC CCS)	1.00	39187.67
2020	Refined liquids (CC)	1.00	187201.12
2020	Refined liquids (steam/CT)	1.00	283989.28
2020	Concentrated solar power	1.00	11052.04
2020	Concentrated solar power with storage	1.00	13714.99
2020	Photovoltaic	1.00	11052.04
2020	Photovoltaic with storage	1.00	13714.99
2020	Wind	1.00	3002.95
2020	Wind_offshore	1.00	3798.48
2020	Wind with storage	1.00	5665.91
2025	Biomass (IGCC CCS)	1.08	8303.27
2025	Biomass (IGCC)	1.05	13137.79
2025	Biomass (conventional CCS)	1.09	7495.74
2025	Biomass (conventional)	1.04	13339.12
2025	Coal (IGCC CCS)	1.08	49780.79
2025	Coal (IGCC)	1.04	256829.06
2025	Coal (conventional pulverized CCS)	1.08	55287.31
2025	Coal (conventional)	1.03	269744.41
2025	Gas (CC CCS)	1.05	29046.52
2025	Gas (CC)	1.02	127801.64
2025	Gas (steam/CT)	1.02	191216.39
2025	Geothermal	NA	29405.90
2025	Hydropower	NA	2148.75
2025	Generation III nuclear	NA	607.98

2025	Generation II nuclear	NA	791.51
2025	Refined liquids (CC CCS)	1.07	36564.56
2025	Refined liquids (CC)	1.02	183328.00
2025	Refined liquids (steam/CT)	1.02	278010.56
2025	Concentrated solar power	NA	10072.71
2025	Concentrated solar power with storage	NA	13260.32
2025	Photovoltaic	NA	10072.71
2025	Photovoltaic with storage	NA	13260.32
2025	Wind	NA	2901.20
2025	Wind_offshore	NA	3662.45
2025	Wind with storage	NA	6088.81
2030	Biomass (IGCC CCS)	1.15	7806.07
2030	Biomass (IGCC)	1.09	12636.63
2030	Biomass (conventional CCS)	1.16	7019.82
2030	Biomass (conventional)	1.07	12891.49
2030	Coal (IGCC CCS)	1.14	47035.09
2030	Coal (IGCC)	1.08	248399.22
2030	Coal (conventional pulverized CCS)	1.15	52008.82
2030	Coal (conventional)	1.06	262907.57
2030	Gas (CC CCS)	1.10	27876.15
2030	Gas (CC)	1.04	125664.49
2030	Gas (steam/CT)	1.04	187870.10
2030	Geothermal	NA	29338.81
2030	Hydropower	NA	2050.38
2030	Generation III nuclear	NA	548.20
2030	Generation II nuclear	NA	695.46
2030	Refined liquids (CC CCS)	1.13	34666.02
2030	Refined liquids (CC)	1.04	179915.80
2030	Refined liquids (steam/CT)	1.04	272278.39
2030	Concentrated solar power	NA	9378.70
2030	Concentrated solar power with storage	NA	12327.47
2030	Photovoltaic	NA	9378.70
2030	Photovoltaic with storage	NA	12327.47
2030	Wind	NA	2743.87
2030	Wind_offshore	NA	3462.08
2030	Wind with storage	NA	5692.64
2035	Biomass (IGCC CCS)	1.21	7406.89
2035	Biomass (IGCC)	1.13	12204.32
2035	Biomass (conventional CCS)	1.22	6675.45
2035	Biomass (conventional)	1.11	12472.94
2035	Coal (IGCC CCS)	1.19	44882.45

2035	Coal (IGCC)	1.11	241015.80
2035	Coal (conventional pulverized CCS)	1.21	49475.05
2035	Coal (conventional)	1.09	256408.73
2035	Gas (CC CCS)	1.14	26938.72
2035	Gas (CC)	1.05	123597.64
2035	Gas (steam/CT)	1.06	184638.92
2035	Geothermal	NA	29326.09
2035	Hydropower	NA	2031.19
2035	Generation III nuclear	NA	534.14
2035	Generation II nuclear	NA	695.46
2035	Refined liquids (CC CCS)	1.18	33197.66
2035	Refined liquids (CC)	1.06	176922.19
2035	Refined liquids (steam/CT)	1.06	267453.20
2035	Concentrated solar power	NA	9196.73
2035	Concentrated solar power with storage	NA	12095.20
2035	Photovoltaic	NA	9196.73
2035	Photovoltaic with storage	NA	12095.20
2035	Wind	NA	2712.69
2035	Wind_offshore	NA	3423.59
2035	Wind with storage	NA	5611.17
2040	Biomass (IGCC CCS)	1.27	7104.16
2040	Biomass (IGCC)	1.16	11860.98
2040	Biomass (conventional CCS)	1.28	6386.27
2040	Biomass (conventional)	1.14	12157.17
2040	Coal (IGCC CCS)	1.24	43106.88
2040	Coal (IGCC)	1.14	235027.83
2040	Coal (conventional pulverized CCS)	1.26	47525.30
2040	Coal (conventional pulverized)	1.11	251325.74
2040	Gas (CC CCS)	1.17	26196.86
2040	Gas (CC)	1.07	121794.76
2040	Gas (steam/CT)	1.07	181956.51
2040	Geothermal	NA	29321.97
2040	Hydropower	NA	2025.21
2040	Generation III nuclear	NA	527.99
2040	Generation II nuclear	NA	687.59
2040	Refined liquids (CC CCS)	1.22	32075.32
2040	Refined liquids (CC)	1.08	174026.58
2040	Refined liquids (steam/CT)	1.08	262143.96
2040	Concentrated solar power	NA	9109.78
2040	Concentrated solar power with storage	NA	11989.35
2040	Photovoltaic	NA	9109.78

2040	Photovoltaic with storage	NA	11989.35
2040	Wind	NA	2701.88
2040	Wind_offshore	NA	3410.55
2040	Wind with storage	NA	5581.46
2045	Biomass (IGCC CCS)	1.31	6861.12
2045	Biomass (IGCC)	1.19	11565.19
2045	Biomass (conventional CCS)	1.33	6142.35
2045	Biomass (conventional)	1.17	11856.99
2045	Coal (IGCC CCS)	1.28	41731.13
2045	Coal (IGCC)	1.16	229794.42
2045	Coal (conventional pulverized CCS)	1.30	45941.12
2045	Coal (conventional pulverized)	1.13	245909.24
2045	Gas (CC CCS)	1.20	25580.47
2045	Gas (CC)	1.08	120235.79
2045	Gas (steam/CT)	1.09	179350.93
2045	Geothermal	NA	29317.60
2045	Hydropower	NA	2018.90
2045	Generation III nuclear	NA	521.72
2045	Generation II nuclear	NA	679.55
2045	Refined liquids (CC CCS)	1.26	31187.42
2045	Refined liquids (CC)	1.09	171500.38
2045	Refined liquids (steam/CT)	1.10	258298.32
2045	Concentrated solar power	NA	9018.11
2045	Concentrated solar power with storage	NA	11441.02
2045	Photovoltaic	NA	9018.11
2045	Photovoltaic with storage	NA	11441.02
2045	Wind	NA	2690.48
2045	Wind_offshore	NA	3396.79
2045	Wind with storage	NA	5113.40
2050	Biomass (IGCC CCS)	1.35	6651.09
2050	Biomass (IGCC)	1.22	11283.80
2050	Biomass (conventional CCS)	1.37	5936.23
2050	Biomass (conventional)	1.19	11571.28
2050	Coal (IGCC CCS)	1.32	40607.93
2050	Coal (IGCC)	1.19	225235.01
2050	Coal (conventional pulverized CCS)	1.34	44664.98
2050	Coal (conventional)	1.15	241741.29
2050	Gas (CC CCS)	1.22	25074.76
2050	Gas (CC)	1.10	118904.06
2050	Gas (steam/CT)	1.10	176818.92
2050	Geothermal	NA	29311.56

2050	Hydropower	NA	2018.90
2050	Generation III nuclear	NA	514.25
2050	Generation II nuclear	NA	669.89
2050	Refined liquids (CC CCS)	1.29	30449.88
2050	Refined liquids (CC)	1.11	169315.66
2050	Refined liquids (steam/CT)	1.12	253951.96
2050	Concentrated solar power	NA	8920.14
2050	Concentrated solar power with storage	NA	11754.62
2050	Photovoltaic	NA	9042.57
2050	Photovoltaic with storage	NA	8920.14
2050	Wind	NA	11754.62
2050	Wind_offshore	NA	3378.50
2050	Wind with storage	NA	5509.63
2015	Biomass (IGCC CCS)	1.00	9951.24
2015	Biomass (IGCC)	1.00	14492.63
2015	Biomass (conventional CCS)	1.00	9025.49
2015	Biomass (conventional)	0.90	14442.35
2015	Coal (IGCC CCS)	0.95	58723.45
2015	Coal (IGCC)	0.90	278915.09
2015	Coal (conventional pulverized CCS)	0.96	66079.70
2015	Coal (conventional)	0.91	287410.29
2015	Gas (CC CCS)	0.96	32732.00
2015	Gas (CC)	0.90	133004.19
2015	Gas (steam/CT)	0.97	199331.67
2015	Geothermal	NA	29492.21
2015	Hydropower	NA	2276.05
2015	Generation III nuclear	NA	687.93
2015	Generation II nuclear	NA	895.55
2015	Refined liquids (CC CCS)	0.91	42919.83
2015	Refined liquids (CC)	0.98	191586.01
2015	Refined liquids (steam/CT)	0.98	291030.34
2015	Concentrated solar power	NA	11005.19
2015	Concentrated solar power with storage	NA	14497.65
2015	Photovoltaic	NA	11005.19
2015	Photovoltaic with storage	NA	14497.65
2015	Wind	NA	3109.28
2015	Wind_offshore	NA	3925.41
2015	Wind with storage	NA	6601.74

Table S12. H_2 production percent yearly breakdown under no carbon price scenario

Scenario	Bio	Technology	Location	2025	2030	2035	2040	2045	2050
Present	With Bio	Biomass gasification CCS	Forecourt production	0.077	0.113	0.137	0.151	0.159	0.154
Present	With Bio	Biomass gasification CCS	Onsite production	0.028	0.012	0.015	0.019	0.021	0.019
Present	With Bio	Biomass gasification CCS	Central	0.021	0.011	0.008	0.007	0.006	0.006
Present	With Bio	Biomass gasification CCS	Central	0.112	0.153	0.127	0.108	0.090	0.075
Present	With Bio	Biomass gasification CCS	Central	0.089	0.155	0.152	0.149	0.147	0.151
Present	With Bio	Biomass gasification CCS	Central	0.002	0.002	0.001	0.001	0.001	0.001
Present	With Bio	Biomass gasification	Forecourt production	0.018	0.009	0.009	0.010	0.011	0.012
Present	With Bio	Biomass gasification	Onsite production	0.001	0.000	0.000	0.000	0.000	0.000
Present	With Bio	Biomass gasification	Central	0.002	0.003	0.003	0.004	0.005	0.006
Present	With Bio	Biomass gasification	Central	0.007	0.010	0.011	0.012	0.015	0.018
Present	With Bio	Biomass gasification	Forecourt production	0.288	0.204	0.218	0.223	0.219	0.209
Present	With Bio	Biomass gasification	Central	0.142	0.117	0.106	0.100	0.094	0.093
Present	With Bio	Coal gasification CCS	Central	0.052	0.076	0.070	0.063	0.057	0.053
Present	With Bio	Coal gasification CCS	Onsite production	0.032	0.012	0.013	0.014	0.013	0.012
Present	With Bio	Coal gasification CCS	Forecourt production	0.003	0.004	0.007	0.010	0.015	0.021
Present	With Bio	Coal gasification CCS	Onsite production	0.000	0.000	0.000	0.001	0.002	0.003
Present	With Bio	Coal gasification CCS	Central	0.099	0.097	0.090	0.085	0.082	0.083
Present	With Bio	Coal gasification CCS	Forecourt production	0.019	0.019	0.027	0.036	0.047	0.060
Present	With Bio	Grid electrolysis	Onsite production	0.007	0.003	0.005	0.009	0.015	0.023
Water+	With Bio	Grid electrolysis	Forecourt production	0.071	0.103	0.120	0.126	0.123	0.108
Water+	With Bio	Grid electrolysis	Onsite production	0.021	0.009	0.010	0.010	0.009	0.006
Water+	With Bio	Grid electrolysis	Central	0.021	0.011	0.008	0.006	0.005	0.004
Water+	With Bio	Grid electrolysis	Central	0.110	0.148	0.120	0.096	0.073	0.054
Water+	With Bio	Grid electrolysis	Central	0.087	0.150	0.143	0.133	0.123	0.118
Water+	With Bio	Natural gas steam ref orming CCS	Central	0.002	0.002	0.001	0.001	0.001	0.001
Water+	With Bio	Natural gas steam ref orming CCS	Forecourt production	0.018	0.008	0.008	0.008	0.008	0.007

Water+	With Bio	Natural gas steam ref orming CCS	Onsite production	0.001	0.000	0.000	0.000	0.000	0.000
Water+	With Bio	Natural gas steam ref orming CCS	Central	0.003	0.005	0.007	0.009	0.011	0.016
Water+	With Bio	Natural gas steam ref orming CCS	Central	0.017	0.025	0.031	0.037	0.045	0.060
Water+	With Bio	Natural gas steam ref orming CCS	Forecourt production	0.265	0.186	0.192	0.187	0.170	0.147
Water+	With Bio	Natural gas steam ref orming	Central	0.140	0.113	0.100	0.089	0.078	0.071
Water+	With Bio	Natural gas steam ref orming	Central	0.051	0.074	0.065	0.057	0.048	0.041
Water+	With Bio	Natural gas steam ref orming	Onsite production	0.023	0.009	0.009	0.008	0.006	0.004
Water+	With Bio	Natural gas steam ref orming	Forecourt production	0.006	0.008	0.015	0.024	0.034	0.047
Water+	With Bio	Natural gas steam ref orming	Onsite production	0.001	0.001	0.001	0.003	0.006	0.009
Water+	With Bio	Natural gas steam ref orming	Central	0.097	0.094	0.085	0.076	0.069	0.065
Water+	With Bio	Nuclear thermal splitting	Forecourt production	0.040	0.042	0.062	0.083	0.105	0.130
Water+	With Bio	Nuclear thermal splitting	Onsite production	0.027	0.012	0.025	0.048	0.086	0.114
Biomass+	With Bio	Nuclear thermal splitting	Forecourt production	0.113	0.159	0.192	0.212	0.224	0.221
Biomass+	With Bio	Nuclear thermal splitting	Onsite production	0.063	0.026	0.032	0.039	0.044	0.041
Biomass+	With Bio	Nuclear thermal splitting	Central	0.019	0.009	0.007	0.005	0.005	0.005
Biomass+	With Bio	Nuclear thermal splitting	Central	0.145	0.195	0.160	0.133	0.109	0.090
Biomass+	With Bio	Solar-power electrolysis	Central	0.077	0.137	0.136	0.135	0.134	0.139
Biomass+	With Bio	Solar-power electrolysis	Central	0.002	0.002	0.001	0.001	0.001	0.001
Biomass+	With Bio	Solar-power electrolysis	Forecourt production	0.017	0.007	0.008	0.008	0.009	0.010
Biomass+	With Bio	Solar-power electrolysis	Onsite production	0.001	0.000	0.000	0.000	0.000	0.000
Biomass+	With Bio	Solar-power electrolysis	Central	0.001	0.002	0.003	0.004	0.004	0.006
Biomass+	With Bio	Solar-power electrolysis	Central	0.006	0.009	0.010	0.011	0.014	0.017
Biomass+	With Bio	Wind-power electrolysis	Forecourt production	0.249	0.168	0.176	0.178	0.175	0.170
Biomass+	With Bio	Wind-power electrolysis	Central	0.124	0.103	0.094	0.089	0.085	0.084
Biomass+	With Bio	Wind-power electrolysis	Central	0.046	0.067	0.061	0.056	0.051	0.047
Biomass+	With Bio	Wind-power electrolysis	Onsite production	0.027	0.009	0.009	0.010	0.009	0.008
Biomass+	With Bio	Wind-power electrolysis	Forecourt production	0.003	0.003	0.005	0.008	0.012	0.017

Biomass+	With Bio	Wind-power electrolysis	Onsite production	0.000	0.000	0.000	0.001	0.001	0.002
Biomass+	With Bio	Grid electrolysis	Central	0.087	0.086	0.081	0.077	0.075	0.077
Biomass+	With Bio	Grid electrolysis	Forecourt production	0.016	0.015	0.021	0.029	0.037	0.049
Biomass+	With Bio	Grid electrolysis	Onsite production	0.006	0.002	0.003	0.006	0.011	0.017
BAU	With Bio	Grid electrolysis	Forecourt production	0.064	0.105	0.128	0.142	0.150	0.147
BAU	With Bio	Grid electrolysis	Onsite production	0.022	0.011	0.014	0.017	0.019	0.018
BAU	With Bio	Grid electrolysis	Central	0.059	0.066	0.057	0.051	0.047	0.044
BAU	With Bio	Grid electrolysis	Central	0.002	0.003	0.003	0.003	0.002	0.002
BAU	With Bio	Grid electrolysis	Central	0.132	0.151	0.137	0.128	0.122	0.125
BAU	With Bio	Grid electrolysis	Central	0.004	0.004	0.002	0.002	0.001	0.001
BAU	With Bio	Grid electrolysis	Forecourt production	0.019	0.011	0.012	0.014	0.015	0.017
BAU	With Bio	Grid electrolysis	Onsite production	0.002	0.000	0.000	0.001	0.001	0.001
BAU	With Bio	Grid electrolysis	Central	0.002	0.004	0.004	0.005	0.006	0.007
BAU	With Bio	Natural gas steam ref orming	Central	0.007	0.011	0.011	0.012	0.013	0.016
BAU	With Bio	Natural gas steam ref orming	Forecourt production	0.333	0.264	0.287	0.296	0.295	0.283
BAU	With Bio	Natural gas steam ref orming	Central	0.273	0.290	0.258	0.235	0.216	0.207
BAU	With Bio	Natural gas steam ref orming	Central	0.013	0.025	0.022	0.020	0.018	0.017
BAU	With Bio	Natural gas steam ref orming	Onsite production	0.024	0.010	0.011	0.013	0.013	0.011
BAU	With Bio	Natural gas steam ref orming	Forecourt production	0.003	0.004	0.007	0.011	0.016	0.022
BAU	With Bio	Grid electrolysis	Onsite production	0.000	0.000	0.001	0.001	0.002	0.003
BAU	With Bio	Grid electrolysis	Central	0.021	0.023	0.020	0.018	0.017	0.017
BAU	With Bio	Grid electrolysis	Forecourt production	0.015	0.016	0.023	0.030	0.039	0.050
BAU	With Bio	Grid electrolysis	Onsite production	0.005	0.002	0.003	0.006	0.010	0.014
Present	w/o Bio	Grid electrolysis	Central	0.117	0.208	0.196	0.186	0.180	0.182
Present	w/o Bio	Grid electrolysis	Central	0.003	0.003	0.002	0.001	0.001	0.001
Present	w/o Bio	Natural gas steam reforming CCS	Forecourt production	0.020	0.011	0.013	0.014	0.015	0.015
Present	w/o Bio	Natural gas steam reforming CCS	Onsite production	0.002	0.000	0.000	0.001	0.001	0.001
Present	w/o Bio	Natural gas steam reforming CCS	Central	0.002	0.003	0.004	0.005	0.006	0.007

Present	w/o Bio	Natural gas steam reforming CCS	Central	0.009	0.013	0.014	0.015	0.017	0.021
Present	w/o Bio	Natural gas steam reforming CCS	Forecourt production	0.370	0.314	0.346	0.355	0.347	0.320
Present	w/o Bio	Natural gas steam reforming CCS	Central	0.188	0.155	0.137	0.125	0.115	0.110
Present	w/o Bio	Solar-power electrolysis	Central	0.069	0.102	0.090	0.081	0.072	0.065
Present	w/o Bio	Solar-power electrolysis	Onsite production	0.049	0.020	0.022	0.023	0.022	0.018
Present	w/o Bio	Solar-power electrolysis	Forecourt production	0.004	0.006	0.011	0.017	0.024	0.032
Present	w/o Bio	Solar-power electrolysis	Onsite production	0.001	0.000	0.001	0.002	0.003	0.004
Present	w/o Bio	Solar-power electrolysis	Central	0.131	0.130	0.116	0.106	0.100	0.099
Present	w/o Bio	Solar-power electrolysis	Forecourt production	0.024	0.029	0.043	0.057	0.074	0.092
Present	w/o Bio	Wind-power electrolysis	Onsite production	0.011	0.005	0.008	0.014	0.024	0.033
Water+	w/o Bio	Wind-power electrolysis	Central	0.115	0.198	0.180	0.161	0.144	0.135
Water+	w/o Bio	Wind-power electrolysis	Central	0.003	0.003	0.002	0.001	0.001	0.001
Water+	w/o Bio	Wind-power electrolysis	Forecourt production	0.019	0.010	0.011	0.010	0.009	0.008
Water+	w/o Bio	Wind-power electrolysis	Onsite production	0.001	0.000	0.000	0.000	0.000	0.000
Water+	w/o Bio	Wind-power electrolysis	Central	0.004	0.007	0.008	0.010	0.013	0.017
Water+	w/o Bio	Solar-power electrolysis	Central	0.022	0.034	0.038	0.043	0.051	0.065
Water+	w/o Bio	Solar-power electrolysis	Forecourt production	0.332	0.272	0.285	0.273	0.242	0.199
Water+	w/o Bio	Solar-power electrolysis	Central	0.183	0.149	0.126	0.108	0.092	0.080
Water+	w/o Bio	Solar-power electrolysis	Central	0.067	0.097	0.083	0.070	0.058	0.047
Water+	w/o Bio	Solar-power electrolysis	Onsite production	0.031	0.013	0.012	0.010	0.008	0.005
Water+	w/o Bio	Solar-power electrolysis	Forecourt production	0.007	0.012	0.022	0.035	0.048	0.062
Water+	w/o Bio	Wind-power electrolysis	Onsite production	0.001	0.001	0.002	0.004	0.007	0.010
Water+	w/o Bio	Wind-power electrolysis	Central	0.128	0.124	0.106	0.092	0.080	0.073
Water+	w/o Bio	Wind-power electrolysis	Forecourt production	0.050	0.063	0.092	0.121	0.148	0.174
Water+	w/o Bio	Wind-power electrolysis	Onsite production	0.036	0.018	0.034	0.062	0.101	0.125
Biomass+	w/o Bio	Wind-power electrolysis	Central	0.117	0.208	0.196	0.186	0.180	0.182
Biomass+	w/o Bio	Wind-power electrolysis	Central	0.003	0.003	0.002	0.001	0.001	0.001

Biomass+	w/o Bio	Biomass gasification	Forecourt production	0.020	0.011	0.013	0.014	0.015	0.015
Biomass+	w/o Bio	Biomass gasification	Onsite production	0.002	0.000	0.000	0.001	0.001	0.001
Biomass+	w/o Bio	Biomass gasification	Central	0.002	0.003	0.004	0.005	0.006	0.007
Biomass+	w/o Bio	Biomass gasification	Central	0.009	0.013	0.014	0.015	0.017	0.021
Biomass+	w/o Bio	Biomass gasification	Forecourt production	0.370	0.314	0.346	0.355	0.347	0.320
Biomass+	w/o Bio	Biomass gasification	Central	0.188	0.155	0.137	0.125	0.115	0.110
Biomass+	w/o Bio	Biomass gasification	Central	0.069	0.102	0.090	0.081	0.072	0.065
Biomass+	w/o Bio	Biomass gasification	Onsite production	0.049	0.020	0.022	0.023	0.022	0.018
Biomass+	w/o Bio	Biomass gasification	Forecourt production	0.004	0.006	0.011	0.017	0.024	0.032
Biomass+	w/o Bio	Biomass gasification	Onsite production	0.001	0.000	0.001	0.002	0.003	0.004
Biomass+	w/o Bio	Biomass gasification	Central	0.131	0.130	0.116	0.106	0.100	0.099
Biomass+	w/o Bio	Biomass gasification	Forecourt production	0.024	0.029	0.043	0.057	0.074	0.092
Biomass+	w/o Bio	Technology	Onsite production	0.011	0.005	0.008	0.014	0.024	0.033
BAU	w/o Bio	Biomass gasification CCS	Central	0.151	0.173	0.158	0.148	0.142	0.144
BAU	w/o Bio	Biomass gasification CCS	Central	0.004	0.005	0.002	0.002	0.001	0.002
BAU	w/o Bio	Biomass gasification CCS	Forecourt production	0.021	0.013	0.015	0.017	0.019	0.020
BAU	w/o Bio	Biomass gasification CCS	Onsite production	0.002	0.001	0.001	0.001	0.001	0.001
BAU	w/o Bio	Biomass gasification CCS	Central	0.002	0.004	0.005	0.005	0.006	0.008
BAU	w/o Bio	Biomass gasification CCS	Central	0.009	0.012	0.013	0.014	0.015	0.018
BAU	w/o Bio	Biomass gasification	Forecourt production	0.395	0.359	0.397	0.411	0.411	0.389
BAU	w/o Bio	Biomass gasification	Central	0.313	0.332	0.296	0.271	0.250	0.239
BAU	w/o Bio	Biomass gasification	Central	0.015	0.028	0.025	0.023	0.021	0.019
BAU	w/o Bio	Biomass gasification	Onsite production	0.036	0.016	0.018	0.020	0.019	0.017
BAU	w/o Bio	Biomass gasification	Forecourt production	0.003	0.006	0.010	0.015	0.022	0.031
BAU	w/o Bio	Biomass gasification	Onsite production	0.001	0.000	0.001	0.002	0.003	0.005
BAU	w/o Bio	Coal gasification CCS	Central	0.024	0.026	0.023	0.021	0.020	0.020
BAU	w/o Bio	Coal gasification CCS	Forecourt production	0.017	0.022	0.032	0.042	0.054	0.068
BAU	w/o Bio	Coal gasification CCS	Onsite production	0.007	0.003	0.005	0.009	0.015	0.020

Table S13. IRA 45V tax incentives for clean H_2 production

Emissions (kg CO_2 kg^{-1} H_2)	>4	>2.5	>1.5	>0.45	<0.45
Tax incentive ($ kg^{-1} H_2)	0	0.6	0.75	1	3

SI References

1. O'Rourke P, Mignone BK, Kyle P, Chapman BR, Fuhrman J, Wolfram P, et al. Supply and Demand Drivers of Global Hydrogen Deployment in the Transition toward a Decarbonized Energy System. Environ Sci Technol. 2023 Dec 5;57(48):19508–18.

2. Ruth M, Jadun P, Gilroy N, Connelly E, Boardman R, Simon AJ, et al. The Technical and Economic Potential of the H2@Scale Hydrogen Concept within the United States [Internet]. 2020 Oct [cited 2023 Nov 16] p. NREL/TP--6A20-77610, 1677471, MainId:29536. Report No.: NREL/TP--6A20-77610, 1677471, MainId:29536. Available from: https://www.osti.gov/servlets/purl/1677471/

3. Argonne National Laboratory. Hydrogen Delivery Scenario Analysis Model [Internet]. 2015 [cited 2025 Feb 11]. Available from: https://hdsam.es.anl.gov/index.php?content=hdsam

4. Wang M, Elgowainy A, Lu Z, Baek K, Balchandani S, Benavides P, et al. Greenhouse gases, Regulated Emissions, and Energy use in Technologies Model ® (2023 .Net) [Internet]. Argonne National Laboratory (ANL), Argonne, IL (United States); 2023 [cited 2025 Feb 9]. Available from: https://www.osti.gov/doecode/biblio/113208

5. Topolski K, Reznicek E, Erdener B, San Marchi C, Ronevich J, Fring L, et al. Hydrogen Blending into Natural Gas Pipeline Infrastructure: Review of the State of Technology [Internet]. 2022 Oct [cited 2023 Dec 30] p. NREL/TP-5400-81704, 1893355, MainId:82477. Report No.: NREL/TP-5400-81704, 1893355, MainId:82477. Available from: https://www.osti.gov/servlets/purl/1893355/

6. EIA. U.S. natural gas consumption set annual and monthly records during 2023 - U.S. Energy Information Administration (EIA) [Internet]. 2024 [cited 2024 Aug 24]. Available from: https://www.eia.gov/todayinenergy/detail.php?id=61923

7. Wei S, Sacchi R, Tukker A, Suh S, Steubing B. Future environmental impacts of global hydrogen production. Energy Environ Sci. 2024 Mar 19;17(6):2157–72.

8. Vallejo V, Nguyen Q, Ravikumar AP. Geospatial variation in carbon accounting of hydrogen production and implications for the US Inflation Reduction Act. Nat Energy. 2024 Dec;9(12):1571–82.

9. Goita EG, Beagle EA, Nasta AN, Wissmiller DL, Ravikumar A, Webber ME. Effect of hydrogen leakage on the life cycle climate impacts of hydrogen supply chains. Commun Earth Environ. 2025 Feb 28;6(1):1–10.

10. Sola A, Rosa R, Ferrari AM. Green Hydrogen and Its Supply Chain. A Critical Assessment of the Environmental Impacts. Adv Sustain Syst. 2025;9(2):2400708.

11. PSE Healthy Energy. Green Hydrogen Proposals Across California [Internet]. 2024 May. Available from: https://www.psehealthyenergy.org/wp-content/uploads/2024/05/Green-Hydrogen-Proposals-Across-California.pdf

12. Kanz O, Brüggemann F, Ding K, Bittkau K, Rau U, Reinders A. Life-cycle global warming impact of hydrogen transport through pipelines from Africa to Germany. Sustain Energy Fuels. 2023;7(13):3014–24.

13. Bertagni MB, Pacala SW, Paulot F, Porporato A. Risk of the hydrogen economy for atmospheric methane. Nat Commun. 2022 Dec 13;13(1):7706.

14. Penev M, Saur G, Hunter C, Zuboy J. H2A Hydrogen Production Model: Version 3.2018 User Guide (DRAFT).

15. O'Rourke PR. 359 Hydrogen and transportation technology update: 2022-02-21 [Internet]. 2022 Feb. Available from: https://jgcri.github.io/gcam-doc/cmp/359-Hydrogen_and_transportation.pdf

16. U.S. Department of Energy's Office of Energy Efficiency and Renewable Energy. NREL Annual Technology Baseline (ATB) Database [Internet]. [cited 2025 May 13]. Available from: https://atb.nrel.gov/archive

17. Pacific Northwest National Laboratory. GCAM v8.2 Documentation: Economic Choice in GCAM [Internet]. [cited 2025 Jul 10]. Available from: https://jgcri.github.io/gcam-doc/choice.html

18. Sampedro J, Waldhoff ST, Edmonds JA, Iyer G, Msangi S, Narayan KB, et al. Residential energy demand, emissions, and expenditures at regional and income-decile level for alternative futures. Environ Res Lett. 2024 Jul;19(8):084031.

19. McFadden D. Conditional logit analysis of qualitative choice behavior. In: Zarembka P, editor. Fontiers in Econometrics. New York: Academic press; 1974. p. 105–42.

20. Clarke JF, Edmonds JA. Modelling energy technologies in a competitive market. Energy Econ. 1993 Apr 1;15(2):123–9.

21. IRS. Federal Register. 2023 [cited 2024 Jun 12]. Section 45V Credit for Production of Clean Hydrogen; Section 48(a)(15) Election To Treat Clean Hydrogen Production Facilities as Energy Property. Available from: https://www.federalregister.gov/documents/2023/12/26/2023-28359/section-45v-credit-for-production-of-clean-hydrogen-section-48a15-election-to-treat-clean-hydrogen

22. Chen W, Yin X, Zhang H. Towards low carbon development in China: a comparison of national and global models. Clim Change. 2016 May 1;136(1):95–108.

23. Wilkerson JT, Leibowicz BD, Turner DD, Weyant JP. Comparison of integrated assessment models: Carbon price impacts on U.S. energy. Energy Policy. 2015 Jan 1;76:18–31.

24. Kaufman N, Barron AR, Krawczyk W, Marsters P, McJeon H. A near-term to net zero alternative to the social cost of carbon for setting carbon prices. Nat Clim Change. 2020 Nov;10(11):1010–4.

25. IEA. Global Energy and Climate Model Documentation 2023 [Internet]. Paris; 2023. Available from: https://www.iea.org/reports/global-energy-and-climate-model

26. Pacific Northwest National Laboratory. GCAM v7.1 Documentation [Internet]. [cited 2025 Feb 11]. Available from: https://jgcri.github.io/gcam-doc/toc.html

27. Congressional Research Service. Credit for carbon oxide sequestration [Internet]. 2023 [cited 2025 Feb 10]. Available from: https://uscode.house.gov/view.xhtml?req=(title:26%20section:45Q%20edition:prelim)
